# Importance of Particle-Phase Reactions in the Growth of Newly Formed Particles

**DOI:** 10.1021/acsearthspacechem.5c00327

**Published:** 2026-02-17

**Authors:** Vignesh Vasudevan-Geetha, Lee Tiszenkel, Zhizhao Wang, Robin Russo, Daniel J. Bryant, Julia Lee-Taylor, Kelley C. Barsanti, Shan-Hu Lee

**Affiliations:** † Department of Atmospheric and Earth Sciences, 14843 University of Alabama in Huntsville, Huntsville, Alabama 35805, United States; ‡ Department of Chemical and Environmental Engineering, 521050 University of California - Riverside, Riverside, California 92521, United States; § Atmospheric Chemistry Observation & Modeling Laboratory, 53593 National Science Foundation National Center for Atmospheric Research, Boulder, Colorado 80307, United States; ∥ Department of Chemistry, 14843 University of Alabama in Huntsville, Huntsville, Alabama 35805, United States; ⊥ Department of Chemistry, 8748 University of York, York YO10 5DD, U.K.

**Keywords:** new particle formation, oxygenated organic molecules (OOM), particle-phase reactions, gas-to-particle conversion, volatility basis set, FIGAERO HrTOF-CIMS, UPLC-ESI Orbitrap mass spectrometer, MS/MS tandem analysis

## Abstract

New particle formation (NPF) is a chemistry-driven process that results in the formation of secondary aerosols and is the main source of global cloud condensation nuclei. Currently, the majority of NPF parametrizations consider volatility-based gas-to-particle conversion of oxygenated organic molecules (OOMs), formed only in the gas phase, and assume thermodynamic equilibrium regardless of aerosol chemical composition or environmental conditions. Here, we performed a comprehensive chemical analysis of the OOMs produced from α-pinene ozonolysis in a fast-flow reactor to elucidate the role of gas- and particle-phase chemistry in the NPF processes. Gas- and particle-phase OOMs were measured with an iodide high-resolution time-of-flight chemical ionization mass spectrometer (HrTOF-CIMS) attached to the filter inlet for gas and aerosol (FIGAERO). Additionally, particle-phase OOMs were detected with off-line ultra-performance liquid chromatography-electrospray ionization (UPLC-ESI) high-resolution Orbitrap MS/MS analysis. Mass spectra of particle-phase OOMs detected with these two methods showed surprisingly similar features, despite entirely different sampling, ionization, and detection techniques. 100% of the OOMs detected with the UPLC-ESI Orbitrap mass spectrometer contained 2–8 isomers with different fragmentation ions. Volatility estimation methods based on elemental composition alone cannot account for chemical functionalities and molecular structures and thus do not differentiate isomers, despite the potential for large volatility differences. Our analysis shows that biogenic OOM dimers can also form directly within the particle phase, via either accretion or decomposition reactions. These particle-phase reactions can affect the chemical composition and volatilities of the OOMs, and, in turn, can affect the phase state and diffusivity of aerosols. Our observations strongly imply the importance of considering gas- and particle-phase chemistry in the growth of freshly formed particles.

## Graphical abstract

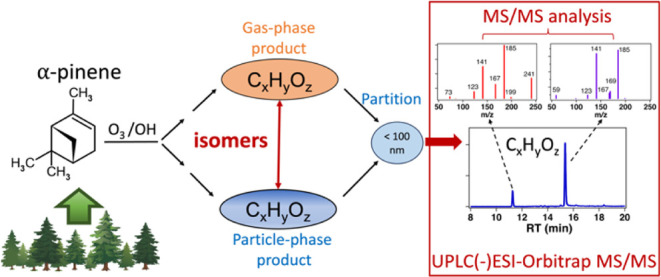

## 1 Introduction

Oxygenated organic molecules (OOMs) formed from oxidation reactions of biogenic volatile organic compounds (BVOCs) can contribute to secondary organic aerosol (SOA) and new particle formation (NPF).^1–5^ In the atmosphere, BVOCs are oxidized by ozone, hydroxyl (OH), and nitrate (NO_3_) radicals to form OOMs. There are many OOMs in the atmosphere with different chemical identities. For example, from ozonolysis of α-pinene alone, thousands of OOMs were detected with different mass-to-charge ratios (*m*/*z*) using a high-resolution time-of-flight chemical ionization mass spectrometer (HrTOF-CIMS), indicating different chemical formulas.^6,7^ Yet molecular structures and formation pathways have been identified for only an extremely limited number of OOMs even for the well-studied α-pinene ozonolysis system.

Dimer OOMs are effective NPF precursors.^6–9^ In the gas phase, OOMs form from reactions involving organic peroxy radicals (RO_2_),^10–12^ as well as stabilized Criegee intermediates (sCI).^13,14^ OOM dimers can also form in the particle phase. For example, OOM dimers form from esterification,^15,16^ Baeyer–Villiger reactions,^17–19^ aldol condensation,^4,20,21^ and diacyl decomposition.^22^ Isomer-resolved characterization of organics has been achieved using liquid chromatography (LC) coupled with high-resolution mass spectrometry, as MS/MS analysis enables the elucidation of molecular structures.^23^ For α-pinene oxidation SOA, C_17_H_26_O_8_ and C_19_H_28_O_7_ dimers have been structurally resolved using a linear ion trap mass spectrometer, and it was shown that they form from acyl trioxide decomposition of a gas-phase product dimer C_19_H_28_O_11._ ^24^ Zhang et al.,^22^ proposed C_17_H_26_O_6_ (*m*/*z* = 309) forms from a diacyl peroxide decomposition reaction, based on the electrospray ionization-quadrupole time-of-flight mass spectrometer (ESI-QTOF-MS) analysis. Using ESI-QTOF-MS, Kristensen et al.^25^ proposed the structure of dimer esters generated from α-pinene ozonolysis in laboratory flow tube experiments, as well as in ambient aerosol samples collected in the boreal forest. Kenseth et al.^18^ demonstrated that both C_19_H_28_O_7_ and C_19_H_30_O_5_ dimers form via particle-phase reactions using authentic standards. In addition to α-pinene oxidation products, MS/MS tandem analysis has also been used to study other monoterpene oxidation products, for example, limonene-derived products using online-nitrate chemical ionization Orbitrap MS/MS,^12^ β-pinene-derived products using LC coupled with ESI-QTOF-MS,^26^ and molecular analysis of SOA derived from monoterpenes such as α-pinene, β-pinene, limonene, 3-carene, and sabinene using LC coupled with an ion trap mass spectrometer.^27^

The current understanding of the NPF processes considers gas-to-particle conversion of low-volatility OOMs.^2,5^ It is generally assumed that OOMs form only in the gas phase, and the OOMs that have sufficiently low volatilities contribute to NPF via gas-to-particle conversion. The volatility (or saturation vapor concentration at a specific temperature) of an OOM moiety is estimated mostly based on the grouped elemental compositions.^9,28–32^ Alternatively, volatilities are also derived from thermogram measurements.^7,33–36^ Laboratory experiments have shown that the volatility estimation method based on the elemental composition overestimates volatilities for monomers and underestimates for dimers, compared to the thermogram method using the filter inlet for gas and aerosol (FIGAERO) attached to an iodide HrTOF-CIMS.^7^

Here, we have analyzed the molecular composition of OOMs formed from the ozonolysis of α-pinene using an ultraperformance liquid chromatography-electrospray ionization Orbitrap mass spectrometer (UPLC/(−)­ESI-Orbitrap MS) and HrTOF-CIMS coupled with a FIGAERO. The chemical composition of OOMs in the gas- and particle-phases was measured simultaneously with FIGAERO HrTOF-CIMS at real time. Based on the LC and high-resolution Orbitrap MS/MS analysis, we propose likely molecular structures and formation pathways of particle-phase OOMs (e.g., C_19_H_30_O_5_ and C_16_H_26_O_6_). To support our experimental interpretations, we also generated the molecular structures of monomer OOMs in the gas phase with the chemically explicit model, the Generator of Explicit Chemistry and Kinetics of Organics in the Atmosphere (GECKO-A),^37–39^ updated per Jenkin et al.^40–42^ We discuss the implications of our findings in the context of NPF processes.

## 2 Materials and Methods

Experiments were carried out in the Tandem Aerosol Nucleation and Growth Environment Tube (TANGENT) setup, which has been used in our previous biogenic and multicomponent NPF studies.^7,43–45^ The data used in this study were generated from our previous biogenic NPF studies by Tiszenkel and Lee;^7^ here, we focus on the chemical composition of OOMs. Briefly, 240 ppb α-pinene in nitrogen was mixed with 1.2 ppm ozone at room temperature (298 K) and dry conditions (RH < 10%). The total residence time in the flow tube was 150 s. The OH radicals present in the experimental system were a byproduct of the monoterpene ozonolysis reaction (thus, a dark OH source). Experiments were conducted without an OH scavenger and without seed aerosols. The peak OH radical concentration was 1.6 ppt, as simulated from the chemical box model using the Master Chemical Mechanism (MCM v3.3.1)^46^ (as described in Tiszenkel and Lee^7^). Box model simulations show that under our experimental conditions, 120 ppb of α-pinene reacted in the TANGENT, with the ratio of α-pinene reacting with ozone versus OH of approximately 2:1. To understand the chemical reaction pathways involving (sCI, we also performed experiments by introducing 10 ppm of formic acid as the sCI scavenger. Aerosol size distributions in the size range from 1 to 200 nm were measured by combining data from a particle size magnifier (PSM; A10, Airmodus)^47^ and a scanning mobility particle sizer (SMPS, which consists of an electrostatic classifier 3080 and a condensation particle counter 3776, both from TSI).

### 2.1 Filter Sample Collection and Extraction

Newly formed aerosol particles from α-pinene ozonolysis were collected at the end of the TANGENT using filters. Whatman glass microfiber filters (Grade GF/D, 2.7 μm pore size, 25 mm diameter) were prebaked at 550 °C for 24 h to remove any residual organics. The particles were collected continuously for 28 h with a 1 LPM (liter per minute) flow through the filter from the experimental tube. Filters were moved to borosilicate glass vials for storage at −20 °C immediately after collection. Three filters were collected from replicate experiments under the same conditions.

Filter samples were divided into quarters, and each quarter was separately extracted by sonicating for 30 min in borosilicate glass vials with 20 mL of methanol (Optima LC/MS, Fisher Scientific) placed in beakers filled with ice. After 30 min, the ice was renewed, and the sample was sonicated for an additional 30 min. The sample was then dried down under a weak stream of pure nitrogen gas in a room-temperature water bath. The dried sample was reconstituted in 2 mL of methanol and filtered through a 0.2 μm nylon syringe filter (Thermo Fisher Scientific) into a sampling vial for LC analysis. Previous studies have shown that organic peroxides decay relatively slowly in 100% methanol.^13,14,48^ Procedural blanks were prepared by subjecting filters that had undergone the prebaking process to the same extraction procedures. LC analysis was performed immediately after filter extraction and reconstitution.

### 2.2 Ultra-Performance Liquid Chromatography/Negative Electrospray Ionization Orbitrap Mass Spectrometry (UPLC/(−)­ESI-Orbitrap MS)

The filter samples were chromatographically separated and analyzed using ultraperformance liquid chromatography (UPLC) (Vanquish, Thermo Fisher Scientific) coupled to an ultrahigh-resolution Orbitrap mass spectrometer (Exploris 120 mass spectrometer, Thermo Fisher Scientific) with an electrospray ionization (ESI) source. The Orbitrap has a mass resolution (*m*/Δ*m*) of 120,000 for MS and 15,000 for MS/MS analysis. The separation of compounds including isomers was achieved using a 100 × 2.1 mm reverse-phase C-18 column with 1.8 μm particle size (Waters, ACQUITY Premier HSS T3). The column and autosampler temperatures were maintained at 40 and 4 °C, respectively. The polar (A) and nonpolar (B) mobile phase solvents were 0.1% formic acid in ultrapure water (Optima LC/MS, Fisher Scientific) and 0.1% formic acid in methanol (Optima LC/MS, Fisher Scientific), respectively. Gradient elution was adopted from Shao et al.^49^ The gradient started with a 1 min postinjection held at 90% A and 10% B, followed by a decrease to 10% A and 90% B over 26 min, then returned to 90% A and 10% B over 2 min, ending with a 2 min column equilibration. The total flow rate was 0.3 mL min^–1^ with an injection volume of 2 μL. The nontargeted mass spectrometric analysis was carried out using optimized ESI parameters: 2.5 kV capillary voltage; 325 °C ion transfer tube temperature; 350 °C vaporizer temperature; and 50, 10, and 1 (arbitrary units) flow rates for sheath gas, auxiliary gas, and sweep gas, respectively. The parent molecules were deprotonated using (−)­ESI and detected as [M–H]^−^ ions; protonated ions by (+)­ESI were detected as [M + H]^+^ and [M + Na]^+^ ions. This study is mostly based on the negative ionization results to directly compare with the particle-phase OOM chemical composition, but we also include some positive MS/MS analysis for additional verification. Nontargeted tandem mass spectrometric (MS/MS) analysis was performed using higher-energy collisional dissociation with a stepped normalized collision energy of 20%, 40%, and 60% for those compounds detected with an ion intensity threshold of 5 × 10^3^. Postacquisition data processing was carried out using a nontargeted compound identification method developed in Compound Discoverer software (version 3.3 SP2, Thermo Fisher Scientific). As outlined in the Introduction, UPLC/(−)­ESI-Orbitrap MS has been typically used in SOA studies (with or without seed particles). In this study, we perform molecular-level characterizations of OOMs formed during NPF processes; however, these new particles may also resemble SOA particles produced without seed particles.

### 2.3 Filter Inlet for Gases and Aerosols on High-Resolution Time-of-Flight Chemical Ionization Mass Spectrometer (FIGAERO HrTOF CIMS)

The chemical composition of OOMs in the gas and aerosol phase was measured with an iodide HrTOF-CIMS (mass resolution of 7,000) attached to FIGAERO (Aerodyne Inc.).^36^ This instrument operation and calibration procedures were described in detail in our previous works.^7,43^ Briefly, during gas-phase sampling, the FIGAERO drew 2 LPM from the flow tube through a stainless steel line onto a Teflon filter (Zerflour, 24 mm diameter, 2 μm pore size, Pall Corp) with an outside diameter of 0.64 cm for 20 min of particle collection. The FIGAERO then directed the filter into a flow of dry, ultrahigh-purity nitrogen for thermal desorption of the collected particles. The nitrogen was heated from room temperature at a rate of 35 °C/min for 5 min to a final temperature of 200 °C. The filter was then heat-soaked with a flow of 200 °C nitrogen for an additional 15 min. The maximum vaporization temperature (*T*_max_) during thermal desorption was used to derive the saturation vapor concentration (C*) of the OOMs based on calibration with standard compounds of known saturation vapor pressures (Figure S1). For calibration, a series of polyethylene glycols (PEGs), azelaic acid, and tricarballylic acid were individually dissolved in acetonitrile at a concentration of 0.05 g L^–1^ and deposited on the FIGAERO filter with a syringe for volatility measurements. The calibration was done at room temperature. The maximum desorption temperature (*T*_max_ in the unit of °C) for each standard compound was plotted against literature values of effective saturation vapor pressure at 300 K (C*, ug/m^3^) (Figure S1). This procedure produced a calibration curve with the following relationship:

1 C*=−0.106Tmax(C°)+7.43

As shown in Figure S1, this calibration curve is consistent with the published results by Ye et al.^31^ However, the current calibration curve deviated from the previous calibration shown in Tiszenkel and Lee,^7^ and the difference was likely caused by the different sample injection methods.^50^ In the present study, we directly injected standard compounds with a syringe, whereas in Tiszenkel and Lee,^7^ an atomizer was used to deposit the standard compounds onto the filter. Using the calibration curve (eq 1), we report the derived effective saturation vapor concentrations of the OOMs. The *T*_max_ is subject to variability depending on the filter mass loading and thermally driven particle-phase chemistry;^51^ however, the present study did not take these effects into consideration.

Combining these two independent high-resolution mass spectrometer techniques, online HrTOF-CIMS and offline UPLC/(−)­ESI-Orbitrap MS, provides a very powerful tool for analyzing the particle-phase chemical composition of the OOMs. So far, only a very few studies have combined these two methods to make molecular-level chemical speciation of OOMs.^51–53^ Each method has different advantages and disadvantages. FIGAERO HrTOF-CIMS measures the particle-phase chemical composition at real time. However, there is the possibility that chemical species (e.g., peroxides, dimers, and large OOMs) are thermally decomposed during the desorption process from the FIGAERO.^7,36,54^ On the other hand, UPLC/(−)­ESI-Orbitrap MS is an offline technique. While this high-resolution MS/MS analysis can provide detailed chemical structure information, artifacts can form during the filter collection, storage, and sample extraction processes.^55–57^ There may be also matrix effects due to relatively high mass concentrations.^58^ Additionally, HrTOF-CIMS and UPLC/(−)­ESI-Orbitrap mass spectrometers can have different ionization efficiencies and detection efficiencies for different chemical species. Despite these differences, these two high-resolution mass spectrometers show very similar chemical compositions of the OOMs, as will be demonstrated in this study (e.g., Figure 1).

**Figure 1:**
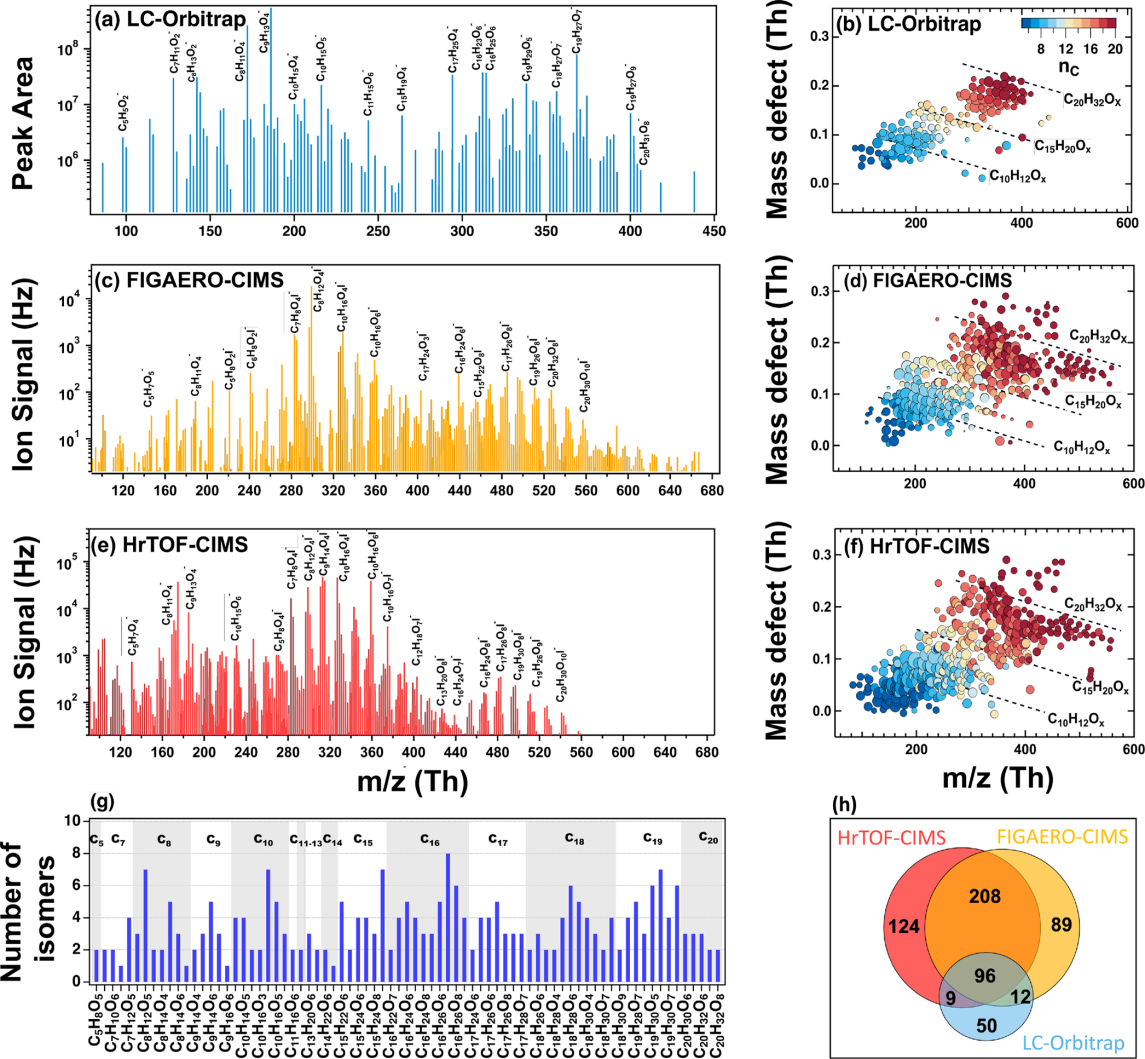
High-resolution mass spectrometer analysis of OOMs detected from α-pinene ozonolysis flow-tube experiment with α-pinene at 240 ppb, ozone at 1.2 ppm, a temperature of 298 K, RH < 10%, [OH] at 1.6 ppt, and a residence time of 150 s. Mass spectra (a, c, and e) and mass defects (b, d, f) of particle-phase OOMs measured with UPLC/(−)­ESI-Orbitrap MS (a and b), particle-phase OOMs measured with FIGAERO iodide HrTOF-CIMS (c and d), and gas-phase OOMs measured with iodide HrTOF-CIMS (e and f). The UPLC/(−)­ESI-Orbitrap MS data show the average peak area across the entire chromatogram. Figure S4 shows the same mass spectra (Figures 1a, 1c, and e) but on a linear scale on the *Y*-axis. (g) The number of isomers identified for each detected OOM in the particle phase with UPLC/(−)­ESI-Orbitrap MS/MS analysis. For clarity, the chemical formulas of only every other OOM are shown here (see Figure 4e for the complete formulas of all the other OOMs). Table S4 lists the selected 77 particle-phase OOMs and their corresponding MS/MS fragmentation ions for each isomer. (h) Venn diagram showing the number of compounds detected from the gas- (red) and particle-phases with FIGAERO HrTOF-CIMS (orange) and from the particle phase with UPLC/(−)­ESI-Orbitrap MS (blue).

### 2.4 Volatility Estimation Based on the Elemental Chemical Composition

Effective saturation vapor concentrations (C*300K in /m^3^) of organic compounds can be estimated based on their grouped elemental chemical composition^9,28–32^ or chemical functionalities.^59–61^ In this study, we used the volatility estimation parametrization (eq 2) adopted from Stolzenburg et al.,^9^ which accommodates hydroperoxide, peroxide, or peroxy-acid functional groups commonly formed in autoxidation and accretion reactions:

2 $$\log_{10}C_{300K}^{*}=\left(n_{C}^{0}-n_{C}\right)b_{C}-n_{O}b_{O}-2\frac{n_{C}n_{O}}{n_{C}+n_{O}}b_{\mathrm{CO}}$$

where $n_{C}^{0}=25,,b_{C}=0.475,,b_{CO}=-0.3$ ; *n*_C_ and *n*_O_ are the number of carbon and oxygen in the OOMs, respectively. The adjusted effect of oxygen *b*_o_ was determined separately for monomers (*b*_o,mon_ = 1.4) and dimers (*b*_o,dim_ = 1.17), because dimers include peroxide bonds, thereby lowering the effect of oxygen on the volatility^9^.

### 2.5 Chemically Explicit GECKO-A Model Simulations

To support the interpretation of the monomeric and dimeric OOMs formed from α-pinene ozonolysis reactions in the gas phase, including their molecular structures, we generated an explicit α-pinene degradation mechanism using GECKO-A,^37–39^ updated as per Jenkin et al.^40–42^ GECKO-A is an automated tool that generates explicit atmospheric oxidation schemes for organic compounds based on experimental data or, in the absence of experimental data, structure–activity relationships (SARs). The GECKO-A-generated mechanisms have been used in many studies to investigate species formed during oxidation under atmospheric conditions.^62–65^ In this study, a five-generation α-pinene oxidation mechanism was generated using GECKO-A and was employed to evaluate the proposed molecular structures of monomer building blocks (as discussed in detail in Section 3.2). The generated scheme includes 870,343 reactions and 152,162 species. Since GECKO-A currently does not include particle-phase reactions, the search and selection processes focused on gas-phase C10 and C9 isomers identified under the typical experimental conditions in TANGENT. Table S1 shows the OOM monomers identified in this study, which match the monomeric products formed in the GECKO-A simulations.

### 2.6 Identifying OOM Dimer Molecular Structures and Possible Formation Pathways

In the present study, we used the following steps to identify the possible chemical structures and formation pathways of dimer OOMs. We have (1) used UPLC-ESI Orbitrap mass spectrometer measurements to identify isomers and their MS/MS fragmentation ions, (2) identified monomer building blocks, based on monomer OOMs detected in the gas- and aerosol-phases from our experiments (e.g., Table S2), (3) used the explicit chemical box model GECKO-A to generate monomeric oxidation products for comparison with our proposed monomeric isomer structures under the same experimental conditions (e.g., Table S1), (4) derived the possible OOM dimer structures, based on the monomer building blocks and relevant chemical reactions available from the current literature (Figure S2 and references cited therein), (5) identified the likely dimer structures that match best the measured MS/MS fragmentation ions, and (6) conducted control experiments, e.g., using the sCI scavenger (formic acid), for additional verification. Even with such a comprehensive procedure, the molecular structures and formation pathways reported in the present study still contain high uncertainties because we did not utilize standard or synthesized chemical compounds to verify the measured liquid chromatograms, MS/MS ion spectra, and the measured molecular structures.^66^

There are thousands of OOMs in the atmosphere. However, there are no commercially available authentic standards for the myriad oxidation products. Studies that have used standards have synthesized them in-house.^18,67–72^ However, this is not a widely available capability. Considering the limited availability of the standard atmospheric samples, currently, identification of molecular structures and formation pathways of the OOMs is one of the most challenging areas in atmospheric chemistry research.

## 3 Results and Discussion

### 3.1 Chemical Composition of OOMs in the Gas and Aerosol Phases

Under the typical experimental conditions, the measured mean diameter of the biogenic new particles was ∼70 nm (see the aerosol size distribution in Figure S3). The corresponding aerosol mass concentration was 135 ± 23 μg m^–3^, calculated from the measured aerosol size distributions, assuming the aerosol density of 1 g cm^–3^. Figure 1 shows the total ion mass spectra and the mass defect plots for the particle-phase OOMs detected with UPLC/(−)­ESI-Orbitrap MS and FIGAERO-HrTOF-CIMS, and gas-phase OOMs detected with the HrTOF-CIMS. For the UPLC/(−)­ESI-Orbitrap MS, we used data that had a signal-to-noise ratio (S/N) larger than 3. For the FIGAERO-HrTOF-CIMS, we used the top 50% OOMs with the highest ion signals (which accounted for about 99% of the total ion signals detected). Both in the gas and particle phases, whether measured with HrTOF-CIMS or UPLC/(−)­ESI Orbitrap mass spectrometer, mass spectra showed the resolved monomers (C_5_–C_10_) and dimers (C_15_–C_20_). In the gas phase, the ratio of ion signals for monomers over dimers was 91:9, whereas in the particle phase, the ratio was 81:19 (both measured with HrTOF-CIMS), indicating that dimers (compared to monomers) are more likely to exist in the particle phase due to relatively lower volatilities, consistent with previous observations.^7^

Remarkably, the particle-phase OOMs spectra taken with UPLC/(−)­ESI-Orbitrap MS and HrTOF-CIMS show strikingly similar patterns. For example, the monomer-to-dimer ratios detected with UPLC/(−)­ESI-Orbitrap MS and HrTOF-CIMS were 70:30 and 81:19, respectively, and the mass-weighted O/C ratios were 0.43 and 0.65, respectively. This agreement is remarkable given the entirely different sampling approaches (offline vs online), ionization techniques (ESI vs chemical ionization), detection methods (Orbitrap mass spectrometer vs HrTOF), as well as potential artifacts introduced during the filter collection, storage, and extraction procedures for LC samples (discussed in Section 2.3). There were substantial numbers of small ions (C_5–6_) detected with the HrTOF-CIMS, likely due to the thermal fragmentation of OOMs during the desorption cycle in the FIGAERO.^7^

Interestingly, the UPLC-ESI Orbitrap MS/MS analysis shows that 100% of the OOMs in the particle phase contain 2–8 isomers (Figure 1g). This result is consistent with previous studies, which showed the presence of isomers in OOMs produced from monoterpene ozonolysis in the laboratory^73^ and OOMs measured in ambient air.^74–76^ Chemical formulas alone, either fitted from HrTOF-CIMS (e.g., mass resolution of 7,000) or derived from the high-resolution Orbitrap mass spectrometer (e.g., mass resolution of 120,000) (when MS/MS analysis is not used), do not differentiate isomers and their molecular structures.

In total, 437 OOMs were identified in the gas phase with the HrTOF-CIMS; 405 OOMs were identified in the particle phase with the HrTOF-CIMS; and 167 OOMs were identified with UPLC/(−)­ESI-Orbitrap MS (Figure 1h). Table S3 shows the OOMs detected in HrTOF-CIMS (gas- and particle-phase) and UPLC/(−)­ESI-Orbitrap MS (particle-phase). There were 124 OOMs that were detected only in the gas phase. 96 OOMs were detected in both the gas (with HrTOF-CIMS) and particle phases (with both HrTOF-CIMS and UPLC/(−)­ESI-Orbitrap MS), suggesting that these lower-volatility OOMs underwent gas-to-particle conversion. In addition, there were 150 OOMs that were detected only in the particle phase (89 detected only with HrTOF-CIMS, 49 only with UPLC/(−)­ESI-Orbitrap MS, and 12 with both instruments).

Table S4 lists 77 selected particle-phase OOMs, along with their distinct retention times (RT) in the liquid chromatogram and different MS/MS fragmentation ions. These 77 compounds were detected in the particle phase by both the HrTOF-CIMS and UPLC/(−)­ESI-Orbitrap MS.

### 3.2 Molecular Structures and Formation Pathways of Particle-Phase OOM Dimers

Using the procedure described in Section 2.6, we propose the possible molecular structures and formation pathways of two unique dimers (C_19_H_30_O_5_ and C_16_H_26_O_6_). C_19_H_30_O_5_ was chosen because it has been identified as a key NPF precursor, as shown by laboratory studies^7,18,48^ as well as field studies in the boreal forest.^25^ C_16_H_26_O_6_ was one of the 12 compounds that were detected only in the particle phase (both with HrTOF-CIMS and UPLC-Orbitrap MS) (Figure 1h and Table S3), consistent with previous studies.^22,51,77^

As discussed in detail below, C_19_H_30_O_5_ likely forms via aldol condensation or esterification, whereas isomers of C_16_H_26_O_6_ likely form from peroxyhemiacetal or decarboxylation reactions. Our results thus demonstrate that OOMs that form exclusively in the particle phase can also contribute to the growth of newly formed particles, consistent with Douverne et al.^78^

#### 3.2.1 C_19_H_30_O_5_ Isomers

The extracted ion chromatogram (EIC) of C_19_H_30_O_5_ ([M–H]^−^ = 337.2019) indicates multiple isomers that eluted at different retention times (RT = 15.3 and 20.2 min) (Figure 2a) with distinctive fragmentation ions (Figure 2b and c) The isomer eluting at RT = 15.6 min had a MS/MS spectrum similar to that at RT = 15.3 min.

**Figure 2:**
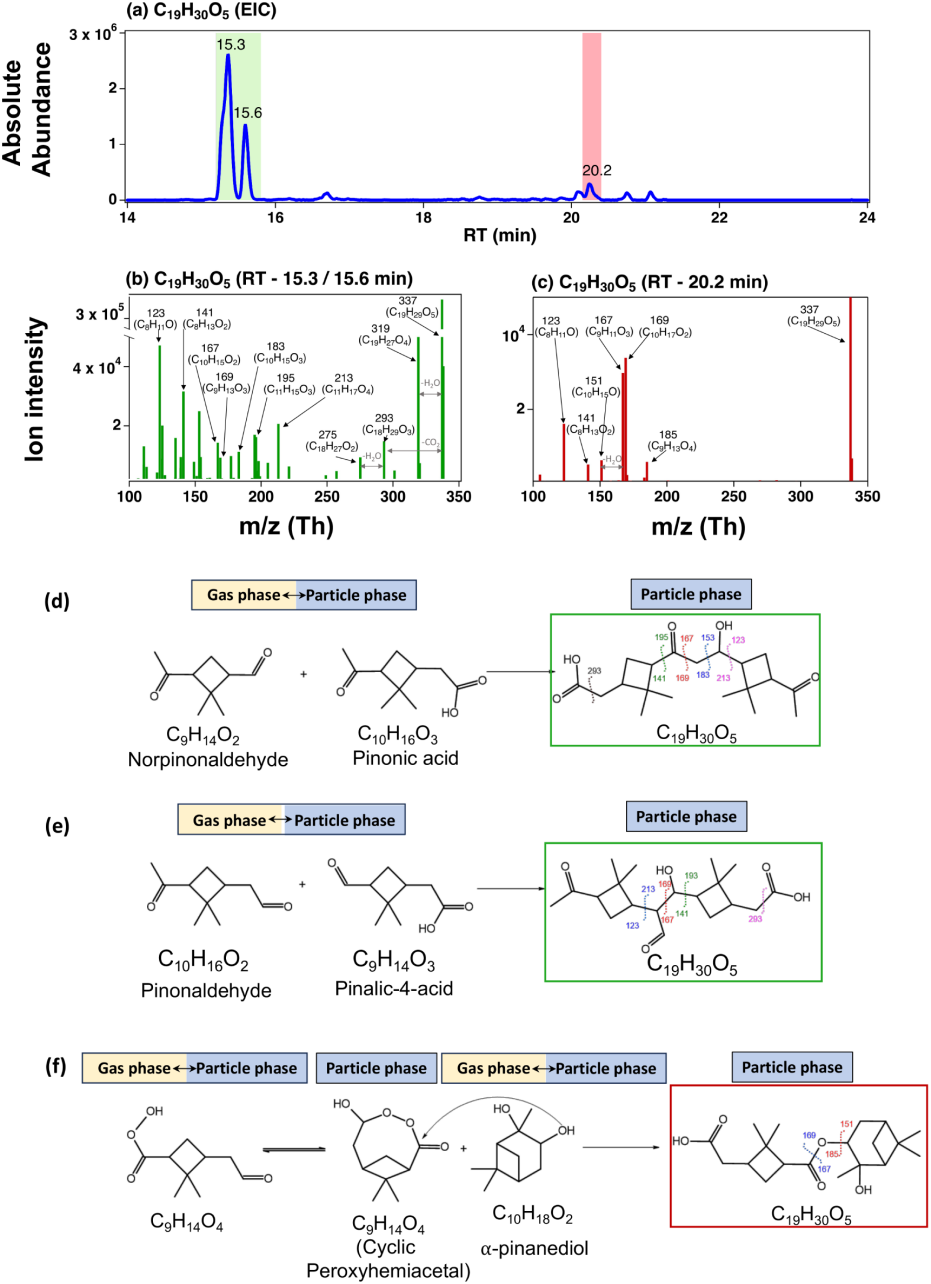
EIC of C_19_H_30_O_5_ (*m*/*z* = 337.2019) (a). MS/MS spectra of the isomers with RT = 15.3 min (b) and 20.2 min (c). The proposed molecular structures and formation pathways for the isomer C_19_H_30_O_5_ with RT = 15.3 min (aldol condensation) (d and e) and RT = 20.2 min (esterification) (f). The latter was adapted from Kenseth et al.^18^ The numbers indicated in the molecular structures are the corresponding nominal *m*/*z* values of [M–H]^−^ fragment ions in the MS/MS spectra; the same for Figure 3.

The fragmentation ions of the first C_19_H_30_O_5_ isomer (RT = 15.3 min, Figure 2b) are in good agreement with those shown by Witkowski and Gierczak.^21^ According to the possible monomer building blocks (Table S2), the aldol condensation product of pinalic-4-acid and pinonaldehyde [Table S2, 4E.1] and *cis*-pinonic acid (C_10_H_16_O_3_) and norpinonaldehyde (C_9_H_14_O_2_) [Table S2, 7D.2] could yield the MS/MS fragments observed here. We propose that aldol condensation from *cis*-pinonic acid and norpinonaldehyde may be responsible for this isomer (Figure 2d). The proposed molecular structure is consistent with MS/MS analysis. The parent ion (*m*/*z* 337) loses H_2_O to form the fragment ion with *m*/*z* 319 and CO_2_ to form *m*/*z* 293, which, in turn, loses another neutral H_2_O molecule to form *m*/*z* 275. The parent ion then undergoes bond cleavages to form ions with *m*/*z* 213/123, 169/167, and 193/141.

Previously, Witkowski and Gierczak^21^ proposed that C_19_H_30_O_5_ forms via the aldol condensation between pinalic-4-acid and pinonaldehyde (Figure 2e). Pinalic-4-acid has been previously identified in the α-pinene ozonolysis system.^21,79^ It is noted that GECKO-A model simulations did not generate pinalic-4-acid (Table S1-monomer 6) because pinalic-4-acid is not formed via a direct oxidation mechanism. However, GECKO-A was able to generate an isomeric pinalic-3-acid (Table S1-monomer 7), which is a common α-pinene oxidation product. Thus, this aldol condensation reaction may be possible (Figure 2e).

Additionally, the secondary ozonide (SOZ) structure formed from the reaction of a stabilized Criegee intermediate (sCI) with norpinonaldehyde (Table S2, 2D.1) can have fragments similar to those observed; however, we did not observe any reduction in the C_19_H_30_O_5_ signals when introducing an sCI scavenger to the α-pinene ozonolysis system.

For the second isomer C_19_H_30_O_5_ (RT = 20.2 min), the MS/MS fragmentation ions (Figure 2c) are consistent with those shown in previous studies.^18,21,25^ Using synthesized standard compounds, Kenseth et al.,^18^ showed that this dimer forms from nucleophilic addition of α-pinanediol ([M + Na]^+^ = 193) to a cyclic acylperoxyhemiacetal formed by the isomerization of *cis*-3-peroxy pinalic acid ([M–H]^−^ = 185), followed by Baeyer–Villiger decomposition (Figure 2f). As illustrated in Figure 2f, there are two main fragmentations near the ester functional group, leading to fragmentation ions with *m*/*z* values of 169/167 and 151/185. The ion with an *m*/*z* of 185 undergoes subsequent fragmentations by losing CO_2_ and H_2_O (neutral loss) to form *m*/*z* values of 141 and 123, respectively. These two monomeric building blocks were identified in the particle phase with UPLC/(−)­ESI-Orbitrap mass spectrometer. The GECKO-A model also predicted the formation of *cis*-3-peroxy pinalic acid and α-pinanediol in the gas phase (Table S1, monomers 13 and 14), with the same molecular structures as shown in Figure 2f. Therefore, it is likely that the C_19_H_30_O_5_ (RT = 20.2 min) isomer forms in the particle phase via esterification reactions, as shown by Kenseth et al.^18^

#### 3.2.2 C_16_H_26_O_6_ Isomers

The EIC of C_16_H_26_O_6_ ([M–H]^−^ *m*/*z* of 313.1677) shows two abundant isomers with the RT at 18.4 and 16.6 min (Figure 3a), with distinctive MS/MS fragmentation ions (Figure 3b and c).

**Figure 3:**
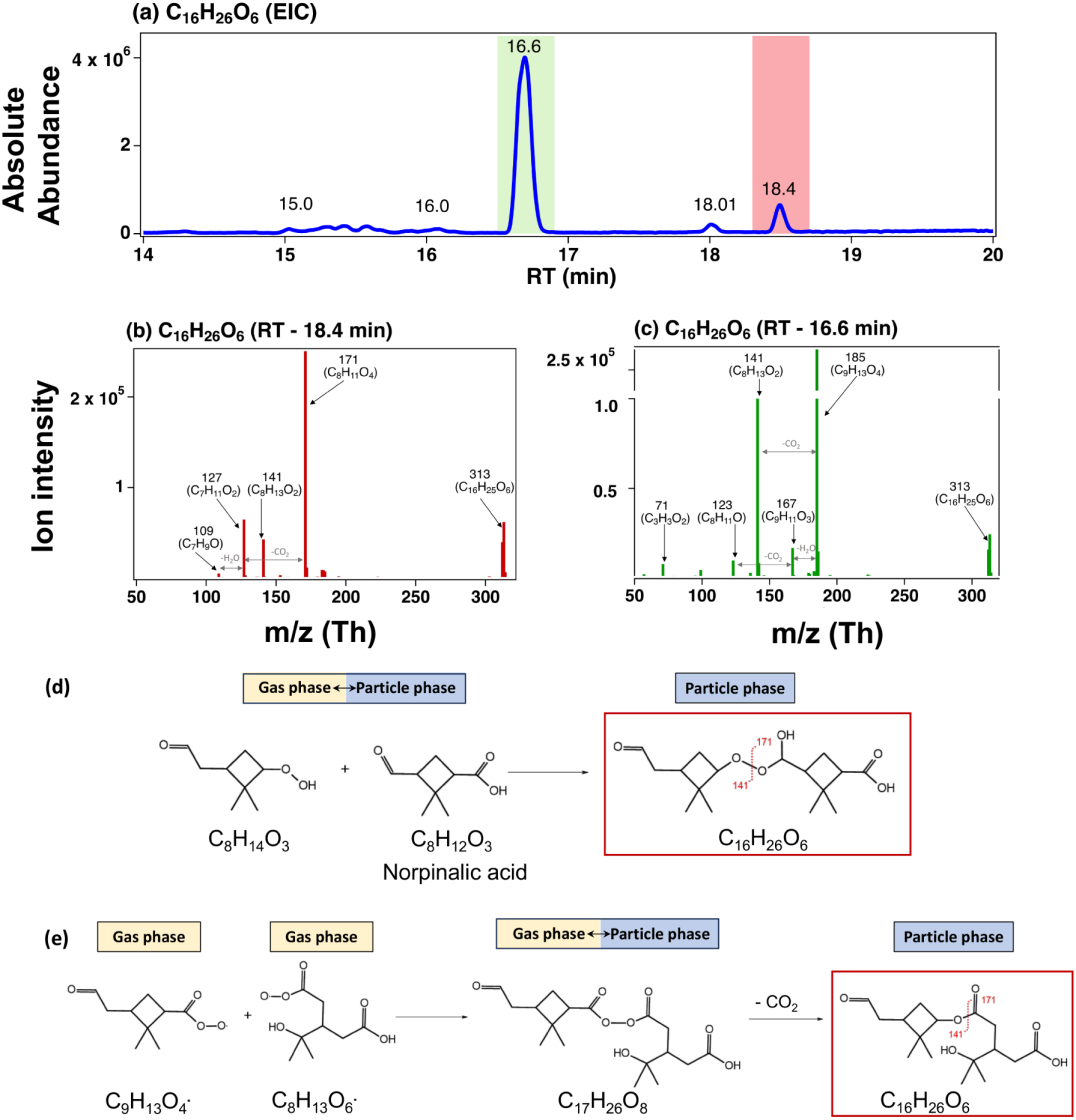
EIC of C_16_H_26_O_6_ (a). MS/MS spectra of two isomers for C_16_H_26_O_6_ with RT = 18.4 and (b) 16.6 min (c). The two possible molecular structures and formation pathways are proposed for C_16_H_26_O_6_ (RT = 18.4 min), peroxyhemiacetal formation (d), and decarboxylation reactions (e). Based on our experimental results, we cannot propose any feasible molecular structures for the C_16_H_26_O_6_ isomer with RT = 16.6 min, as discussed in the text.

Fragmentation of the C_16_H_26_O_6_ (RT = 18.4 min) isomer yields daughter ions with nominal [M–H]^−^ *m*/*z* of 171 (C_8_H_11_O_4_^–^), 141 (C_8_H_13_O_2_^–^), 127 (C_7_H_11_O_2_^–^), and 109 (C_7_H_9_O^–^) (Figure 3b). The fragmented ions with *m*/*z* 171, 129, and 109 can be attributed to two of the most abundant monomers: terpenylic acid (C_8_H_12_O_4_, MW 172) and *cis*-norpinic acid (C_8_H_12_O_4_, MW 172) produced in the α-pinene ozonolysis system.^16,26^ The ions with *m*/*z* = 127 and 109 are the subsequent fragments of *m*/*z* = 171 formed by the loss of CO_2_ and H_2_O, respectively. The daughter ion C_8_H_13_O_2_^–^ ([M–H]^−^ = 141) forms by the loss of CO_2_ from C_9_H_14_O_4_ (MW = 186). Therefore, the possible monomer building blocks for C_16_H_26_O_6_ are C_8_H_13_O_2_^–^ (*m*/*z* 141), which is the fragmentation ion of pinic acid and C_8_H_11_O_4_^–^ (*m*/*z* 171). C_8_H_11_O_4_^–^ (*m*/*z* 171) can be a fragmentation ion of either *cis*-norpinic or terpenylic acid. Because *cis*-norpinic acid has two carboxylic acid moieties, it can either undergo esterification or react with sCI in the gas phase to form α-acyloxyalkyl hydroperoxides.^79^ However, the esterification reaction for C_16_H_26_O_6_ is less likely because another closed-shell monomeric building block C_8_H_16_O_3_ was not detected. Reaction with sCI is also ruled out because we did not observe any signal reduction for C_16_H_26_O_6_ (RT = 18.4 min) when the sCI scavenger was added. Thus, the monomer with *m*/*z* of 171 is more likely from terpenylic acid (*m*/*z* of 171).

Based on the above analysis, we propose two possible structures for C_16_H_26_O_6_ (RT = 18.4 min; Figure 3d and e), which can yield the MS/MS fragmentation ions (Figure 3b). First, C_16_H_26_O_6_ may be a peroxyhemiacetal formed from norpinalic acid (C_8_H_12_O_3_) and C_8_H_14_O_3_ that has a hydroperoxide moiety (Figure 3d). Second, it is also possible that C_16_H_26_O_6_ forms from particle-phase decarboxylation of the diacyl dimer C_17_H_26_O_8_, which forms from gas-phase RO_2_–RO_2_ dimerization of C_8_H_13_O_6_• (a terpenylic acid precursor RO_2_ radical^80^) and C_9_H_13_O_4_• (the second-generation RO_2_ radical from α-pinene ozonolysis) (Figure 3e). The second proposed structure is also in agreement with the HrTOF-CIMS detection of the RO_2_ + RO_2_ termination products of C_8_H_14_O_5_ and C_9_H_14_O_3_ from C_8_H_13_O_6_^•^ and C_9_H_13_O_4_^•^ RO_2_ radicals, respectively. The GECKO-A model simulated the monomer building blocks required in the above two proposed reactions (Table S1, monomers 17–20). The proposed mechanism is consistent with previous observations that a major fraction of C_14–18_ dimer formation in α-pinene ozonolysis involves acyl-RO_2_ monomer.^81^ Both of these structures can explain the observed negative mode MS/MS spectra of C_16_H_26_O_6_ (RT = 18.4 min). The positive mode MS/MS spectra of this isomer show an additional peak of C_8_H_14_O_5_ ([M + Na]^+^ *m*/*z* of 213.0732) (Figure S5a), which could be a fragmented ion from the molecular structures of C_16_H_26_O_6_.

As for C_16_H_26_O_6_ with RT = 16.6 min, at present, we cannot propose any feasible molecular structures and formation pathways based on our observations and published literature. The fragmentation of C_16_H_26_O_6_ (Figure 3c) is in good agreement with those shown by Kristensen et al.^25^ It yields daughter ions with nominal *m*/*z* values of [M–H]^−^ of 185, 167, 141, 123, 71, and 57. The pattern of daughter ions is similar to that of pinic acid fragmentation (C_9_H_14_O_4_, MW = 186), suggesting that the monomer building blocks consist of C_9_H_14_O_4_ and C_7_H_12_O_2_. While the C_7_H_12_O_2_ fragment was not detected in the negative mode ([M–H]^−^), in the positive mode [M + Na]^+^ MS/MS spectrum, there were both C_9_H_14_O_4_ and C_7_H_12_O_2_ (Figure S5b). These fragmentation ions suggest that this C_16_H_26_O_6_ isomer has the same molecular structure as shown in Zhang et al. (2015)^22^ and may form from particle-phase diacyl peroxide decomposition. As stated above, C_16_H_26_O_6_ was not detected in the gas phase. However, C_17_H_26_O_8_ was detected in both the gas and particle phases. One possibility is that the isomer of C_16_H_26_O_6_ (RT = 16.6 min) forms in the particle phase from a diacyl peroxide (C_17_H_26_O_8_) via diacyl peroxide decomposition. The precursor C_17_H_26_O_8_ can form in the gas phase from C_9_H_13_O_5_• acetylperoxy radical and a ring-opening acetylperoxy radical (C_8_H_13_O_5_•) via the RO_2_ + RO_2_ dimerization, and then subsequently partition into the particle phase (Figure S6). However, C_8_H_13_O_5_^•^ was not detected with CIMS, and thus, we excluded this possible molecular structure and formation pathway.

### 3.3 Volatilities of OOMs Estimated from FIGAERO Thermogram vs Elemental Composition Contribution

We compared the effective saturation vapor concentrations at 300 K (C*) of the OOMs derived from FIGAERO thermogram measurements and those estimated based on a group contribution method based on elemental composition (eq 2).^9^ As shown in Figure 4, the log_10_ C* (μg/m^3^) values range from −5 to +5, encompassing the extremely low-volatility organic compounds (ELVOC) through intermediate-volatile organic compounds (IVOC) ranges (Figure 4a and b). Both methods capture the general trend of decreasing saturation vapor concentration with increasing molecular weight and increasing O/C ratios (Figure 4c and d). Interestingly, both methods also show the “teeth-like” feature observed in measured saturation vapor concentrations with increasing molecular weight, oxygen, and carbon numbers, as predicted from the group contribution method (Figure 4e).

**Figure 4:**
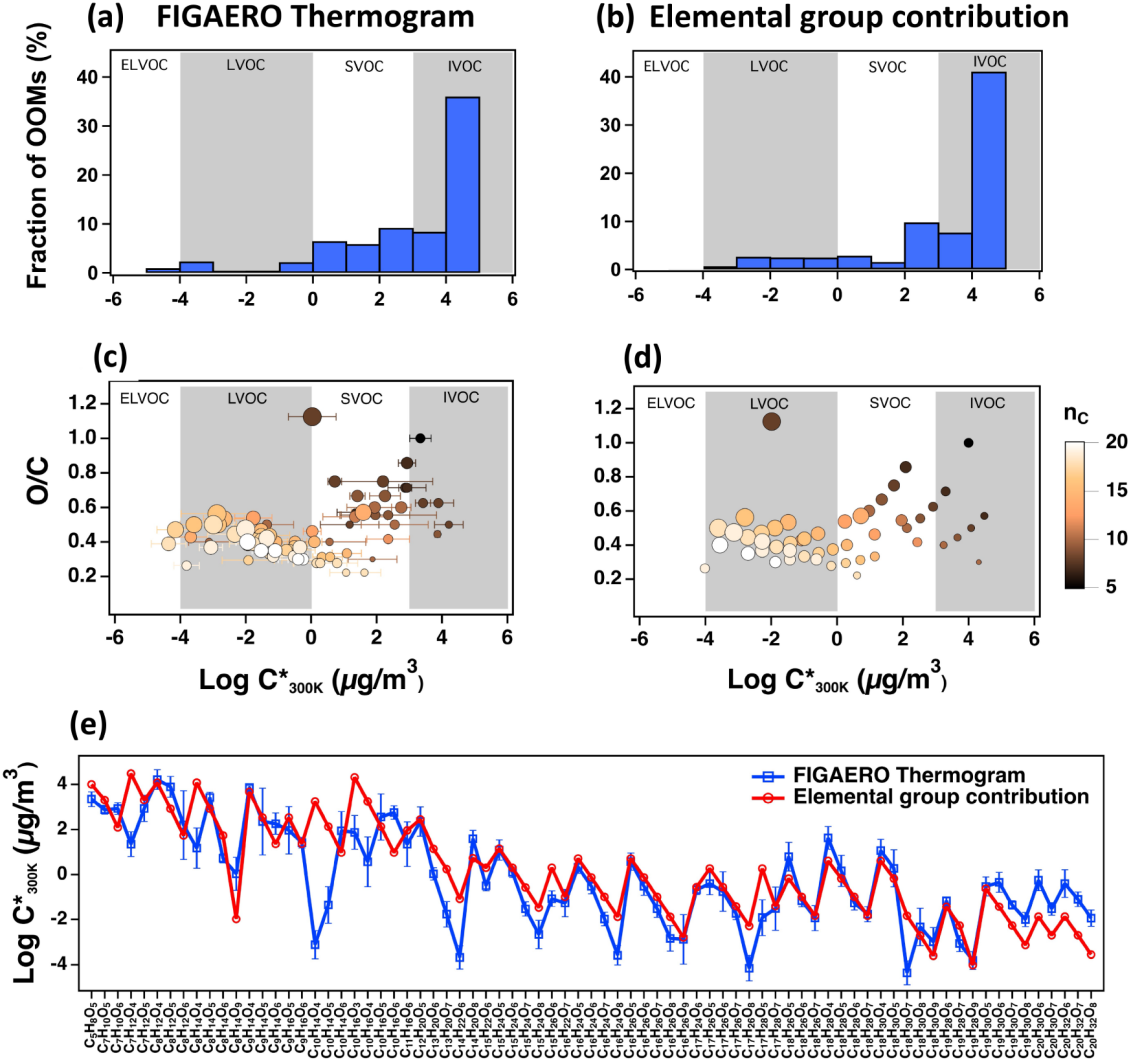
Volatility bins based on the logarithms of effective saturation vapor concentrations of OOMs at 300 K (log C*_300 K_ in μg/m^3^) derived from the FIGAERO thermogram measurements (a) and the volatility estimation method based on the elemental composition adopted from Stolzenburg et al.^9^ (eq 2) (b). The volatility ranges, ELVOC, LVOC, SVOC, and IVOC, are indicated. O/C ratios vs log_10_ C*_300K_ derived from FIGAERO thermogram (c) and the volatility estimation method based on the elemental composition^9^ (eq 2) (d). Log_10_ C*_300K_ values of the two methods are compared for different OOM chemical formulas in Figure 1e. The horizontal (Figure 4c) and vertical bars (Figure 4e) indicate one standard deviation of the FIGAERO-measured volatilities from three replicate measurements.

There are also several discrepancies between the volatilities estimated based on elemental composition and thermograms. In general, the volatility estimation method based on elemental composition underestimates the OOM fractions in the ELVOC and SVOC volatility bins but overestimates the OOM fraction in the LVOC and IVOC bins relative to the thermograms (Figure 4a and b). FIGAERO measurements also show that the C_16–17_ compounds have the lowest measured volatility, whereas the volatilities based on the elemental composition show that C_20_ OOMs are the least volatile (Figure 4c and d). In fact, volatilities based on elemental composition are lower than those derived from the FIGAERO measurement by roughly 2 orders of magnitude for C_20_ OOMs. The volatilities estimated based on the elemental composition show decreasing volatility with increasing oxygen number (e.g., for C_10_H_14_O_4–6_). On the other hand, the FIGAERO measurement showed an increase in volatility from 4 to 6 oxygen atoms in line with the observations of Kurtén et al. (2016),^82^ and this trend could be due to the intramolecular hydrogen bonding.

Another notable observation is related to hydrogen atoms (Figure 4e). Currently, hydrogen is not considered in elemental composition-based volatility estimations (e.g., eq 2). For compounds with a constant number of carbon or oxygen atoms but differing hydrogen numbers (e.g., C_8_H_12,14_O_6_ or C_9_H_14,16_O_6_) the saturation vapor concentrations derived from the thermogram tend to be higher by 1–3 orders of magnitude for the compounds containing fewer hydrogen, as shown in thermogram measurements (Figure 4e). On the other hand, dimers such as C_19_H_28,30_O_7_ and C_19_H_28,30_O_6_ show the opposite trend. These observations can be explained by the hydrogen-bonding effects on the saturation vapor pressures, as predicted in COSMOtherm.^82,83^ COSMOtherm shows that, in the case of monomers, an increase in intermolecular hydrogen bonding by hydrogen-donor functional groups likely decreases the volatility, whereas dimers with more hydrogen-donor groups can favorably form intramolecular hydrogen bonds that exceed the effect of the increasing number of carbons or oxygens on saturation vapor pressures.

Hydrogen bonding, functional groups, and structural differences between isomeric compounds can affect volatilities,^61,84,85^ but these attributes are not considered in the volatility estimation method based on the elemental composition (e.g., eq 2).^9^

### 3.4 Importance of Considering Molecular Structures and Particle-Phase Reactions in NPF

The volatility basis set (VBS) framework was developed to simplify the model representation of a large number of atmospherically relevant organic compounds (e.g., thousands), particularly in models of SOA, by binning compounds into decadally spaced volatility bins based on effective saturation vapor concentrations.^29,86,87^ The volatility bins are assigned volatility categories from ULVOC (ultra-low volatile organic compounds), ELVOC, LVOC, SVOC, IVOC, to VOCs based on their log C* (300 K) in μg/m^3^ (e.g., Figure 4). Saturation vapor concentrations have been estimated using the number of C, O, and N atoms,^9,28–32^ and more recently also including S atoms.^88^ It should be noted that there are other volatility estimation methods that are based on chemical composition but also include chemical functionalities.^59–61^

In recent years, volatility estimation based on elemental composition^9,28–32^ has been adopted into NPF parametrizations. These NPF parametrizations consider gas-to-particle conversion of OOMs formed only in the gas phase, based on volatility estimated from elemental composition alone. These parametrizations neglect the contribution of OOMs formed in the particle phase and assume that aerosol particles are liquid, which allows instant diffusion of chemical species in the condensed phase.^6,9,30,31,89–91^ With these simplifications, these parametrizations are unable to capture the effects of molecular structure on volatility and phase state on time scales for gas-to-particle conversion, as well as potential contributions of particle-phase reactions in forming OOMs that contributeto NPF.^78^ Our results demonstrate that there is a wide range of OOMs likely contributing to the growth of newly formed particles with a diversity of isomers and formation in the gas and particle phase. Neglecting the contribution of particle-phase OOMs and not accounting for the range of molecular structures on volatilities can lead to an underestimation or overestimation of the contribution of organic compounds to particle growth rates.

Here, an analogy is given with sulfuric acid to explain the importance of chemistry (compared to volatility) in NPF. Sulfuric acid is the most important nucleation precursor.^5,92,93^ However, the saturation vapor pressure at 298 K is 1.3 × 10^–3^ Pa^94,95^ which translates to its log C* (μg/m^3^) of 1.71. Thus, its volatility is in the SVOCs range, a significantly higher volatility than ELVOC/ULVOC biogenic OOM dimers, trimers, or tetramers (also see Figure 4).^6–8^ Despite its moderate volatility, sulfuric acid is still the most important nucleation precursor in the atmosphere. This is *not* because of its volatility, but because sulfuric acid molecules have a unique molecular structure, which is very ideal for the formation of intermolecular hydrogen bonding between sulfuric acid molecules, as well as between sulfuric acid and water molecules in the atmosphere. These hydrogen bondings efficiently stabilize critical clusters in the atmosphere and allow them to grow larger efficiently. Also, sulfuric acid can be deprotonated in the particle phase and so becomes effectively nonvolatile. Therefore, in the real atmosphere, almost 100% of NPF events observed in the atmosphere are initiated by sulfuric acid.^5,93^ The typical noontime peak sulfuric acid concentrations are mostly at the 10^6^ cm^–3^ level even during the NPF events.^5,43,96^ NPF due to pure biogenic OOMs without sulfuric acid has been observed extremely rarely,^2^ despite exceedingly lower volatilities (ELVOC and ULVOC) of OOM dimers (their ambient concentrations are comparable to, or even higher than, sulfuric acid^43^).

The same analysis can also be applied to ammonia, another key nucleation precursor.^5,92,93^ The volatility of ammonia is relatively high (1 × 10^6^ Pa at 298 K);^97,98^ thus, log C* (μg/m^3^) is 9.83 (within the VOC range). Still, strong acid–base reactions between sulfuric acid and ammonia make ammonia an essential nucleation precursor. Thus, considering only volatilities in nucleation (without chemistry) is inadequate because nucleation is not just a simple gas-to-particle conversion of a mass of low-volatility chemical species. Rather, nucleation is the process in which thermodynamically stable clusters form from gas phase species.^5,92^ In other words, chemistry matters greatly in NPF. This is the fundamental difference between NPF (driven by chemistry and volatility) and SOA formation (mostly driven by volatility, except for multiphase or aqueous reactions).

In the case of organics, not only the C, O, and N numbers but also chemistry plays important roles in aerosol nucleation and growth. For example, specific chemical functional groups (e.g., carboxylic acid) can form hydrogen bonding with sulfuric acid to stabilize critical clusters and enhance nucleation rates,^99^ despite their relatively high volatilities (e.g., especially for small aromatic acids). Thus, the VBS framework, solely based on the elemental composition, is insufficient for NPF parametrizations.

As shown in the present study, 100% of the particle-phase OOMs detected with the UPLC-ESI Orbitrap mass spectrometer contain isomers (Figure 1e), with distinctive MS/MS fragmentation ions (Figures 2 and 3), indicating different molecular structures and chemical functional groups. However, the effective saturation vapor concentrations calculated based on the elemental composition cannot distinguish the differences in volatilities for different isomers. As shown in the present study (Figure 4) as well as in other studies,^7^ there exist discrepancies in volatilities estimated based on the elemental composition and thermogram (Figure 4), and these different volatilities may predict NPF differently. Future studies are required to understand the extent to which different isomers affect the volatilities (and volatility bins). These different molecular structures affect not only the volatility but also the diffusivity in the condensed phase. Very importantly, different molecular structures and functional groups determine the ability to nucleate and grow, for example, via hydrogen bonding (as discussed above).

Our results demonstrate that newly formed biogenic particles contain not only OOMs that originate from gas-to-particle conversion but also OOMs that form exclusively in the particle phase via accretion or decomposition reactions (Figures 2 and 3). These results demonstrate that condensation of gas-phase precursors alone cannot account for the chemical composition and concentration of the OOMs present in the newly formed particles. Whether through accretion or decomposition, particle-phase reactions not only affect the volatilities of OOMs but also the diffusivity and phase state of aerosol particles.^300^

Currently, measured particle growth rates under most atmospheric conditions cannot be explained with the measured chemical precursors including gas-phase^104^ Our observations strongly imply that the lack or oversimplification of chemistry in the NPF parametrization can be attributed to this discrepancy. For example, particle-phase reactions (accretion or decomposition) of OOMs can directly affect particle growth or shrinkage processes via formation of additional OOMs (other than those from gas-to-particle conversion) but also by changing volatility and diffusivity. Additionally, volatility calculations of the OOMs based on the grouped elemental compositions fail to account for molecular structural information.

## 4 Conclusions

We have investigated the chemical composition of gas- and particle-phase OOMs generated from the ozonolysis of α-pinene using two high-resolution mass spectrometer methods, HrTOF-CIMS with the FIGAERO inlet and UPLC/(−)­ESI-Orbitrap MS, to identify their potential molecular structures and formation pathways. The first key result of this study is the confirmation of the formation of OOMs within the particle phase during NPF from biogenic precursors. For example, we confirmed that C_19_H_30_O_5_ isomers form via aldol condensation^21^ or esterification,^18^ as previously identified during these cited SOA studies. Additionally, we propose that isomers of C_16_H_26_O_6_ form from peroxyhemiacetal or decarboxylation reactions. The second key result is that 100% of the particle-phase OOMs produced from the ozonolysis of α-pinene are isomeric, with 2–8 isomers each, as shown by the UPLC/(−)­ESI-Orbitrap MS/MS analysis.

Our experimental results strongly imply the importance of considering molecular structures and particle-phase reactions in the NPF processes. Future studies are required to understand how particle-phase reactions (accretion or decomposition) of OOMs affect the growth and shrinkage of new particles under different atmospheric conditions. Updated volatility estimation methods that account for the molecular structural information need to be incorporated into the NPF parametrizations to accurately identify the effective nucleation and growth precursors.

## Supplementary Material



## Data Availability

The data underlying this study are openly available on Zenodo at 10.5281/zenodo.17410443.

## Notes

FIGAERO thermogram calibration results (Figure S1); an overview of known OOM dimer reaction pathways (Figure S2); a particle number size distribution in TANGENT (Figure S3); linear scale mass spectra of OOMs detected (Figure S4); a positive mode MS/MS spectrum and structure (Figure S5); C_16_H_26_O_6_ formation mechanism (Figure S6); GECKO-A results (Table S1); monomer building blocks and dimer structure of C_19_H_30_O_5_ (Table S2); a list of detected compounds by HrTOF-CIMS and UPLC/(−)­ESI-Orbitrap MS (Table S3); isomers of OOMs and their major MS/MS fragments identified by UPLC/(−)­ESI-Orbitrap MS (Table S4) (PDF)
