# Supplementary material for: Importance of Particle-Phase Reactions in the Growth of Newly Formed Particles
Source: ACS Earth Space Chem. 2026 Feb 17;10(3):697–710. doi: 10.1021/acsearthspacechem.5c00327 (PMC13007022; doi:10.1021/acsearthspacechem.5c00327)
Supplement: Supplementary file 1 [file sp5c00327_si_001.pdf]

Supporting Information

Importance of Particle-Phase Reactions in the Growth of Newly Formed Particles

Vignesh Vasudevan-Geetha[1], Lee Tiszenkel[1], Zhizhao Wang[2,3], Robin Russo[4], Daniel J. Bryant[5], Julia Lee-Taylor[3], Kelley C. Barsanti[3], Shan-Hu Lee[1,4*]

[1]Department of Atmospheric and Earth Sciences, University of Alabama in Huntsville, Huntsville, AL 35805, United States

[2]Department of Chemical and Environmental Engineering, University of California - Riverside, Riverside, CA 92521, United States

[3]Atmospheric Chemistry Observation & Modeling Laboratory, National Center for Atmospheric Research, Boulder, CO 80307, United States

[4]Department of Chemistry, University of Alabama in Huntsville, Huntsville, AL 35805, United States

[5]Department of Chemistry, University of York, York, YO10 5DD, United Kingdom

*Corresponding author (shanhu.lee@uah.edu)

Figures S1 to S6

Tables S1 to S4

Supplement references

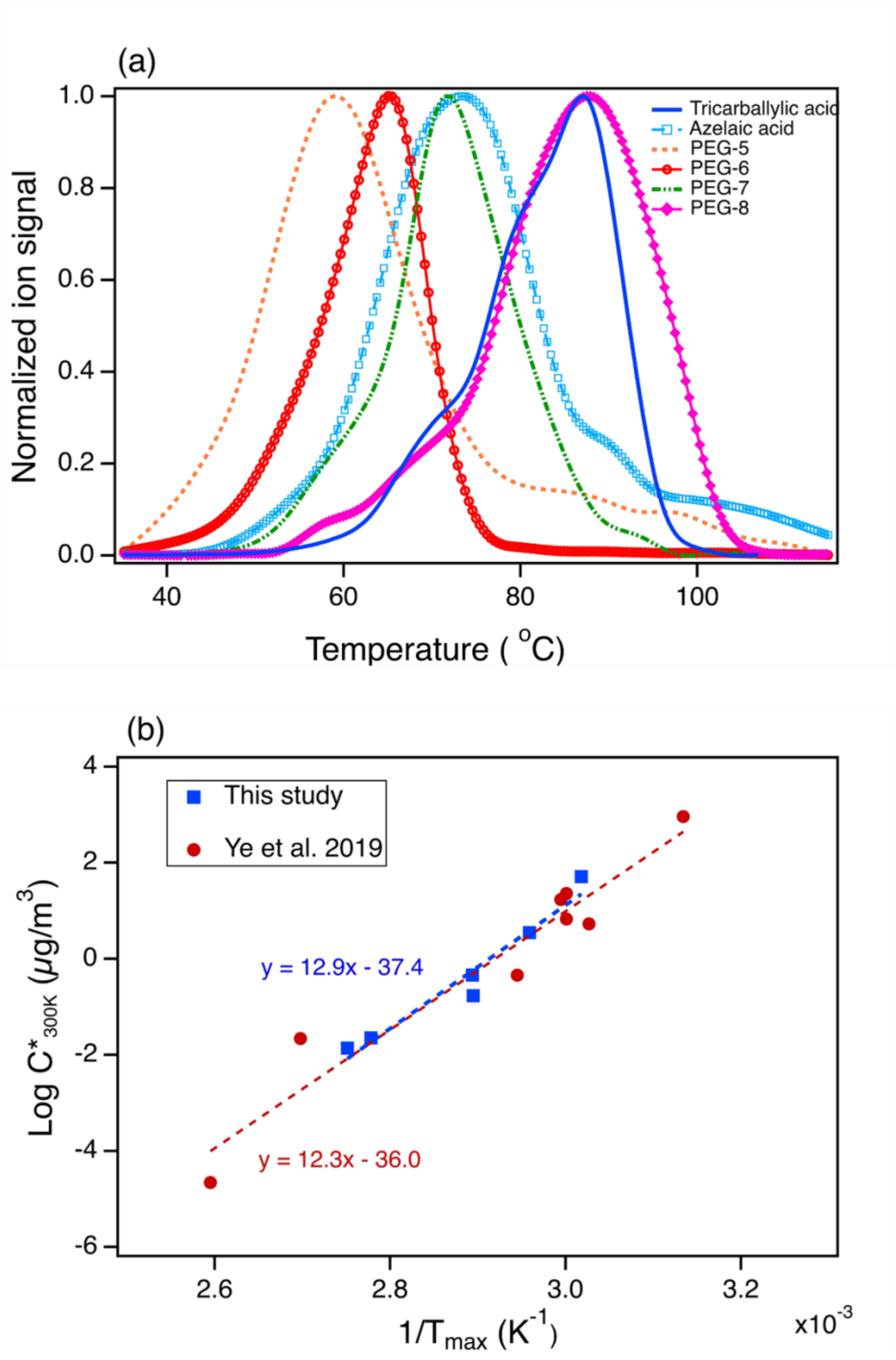

Figure S1. FIGAERO thermogram calibration results obtained with the standard homologs of polyethylene glycol (PEG 5-8), tricarballylic acid, and azelaic acid. (a) Normalized thermogram signals taken during each standard compound's desorption cycle. Desorption temperatures (T_{max}) is derived from these measurements and used in the calibration curve. (b) The logarithms of

effective saturation vapor concentrations of OOMs at 300 K (Log C^*_{300K}) for standard calibration compounds vs. $1/T_{max}$ (blue squared). In comparison, the calibration results of Ye et al.,[1] are also included (red circles).

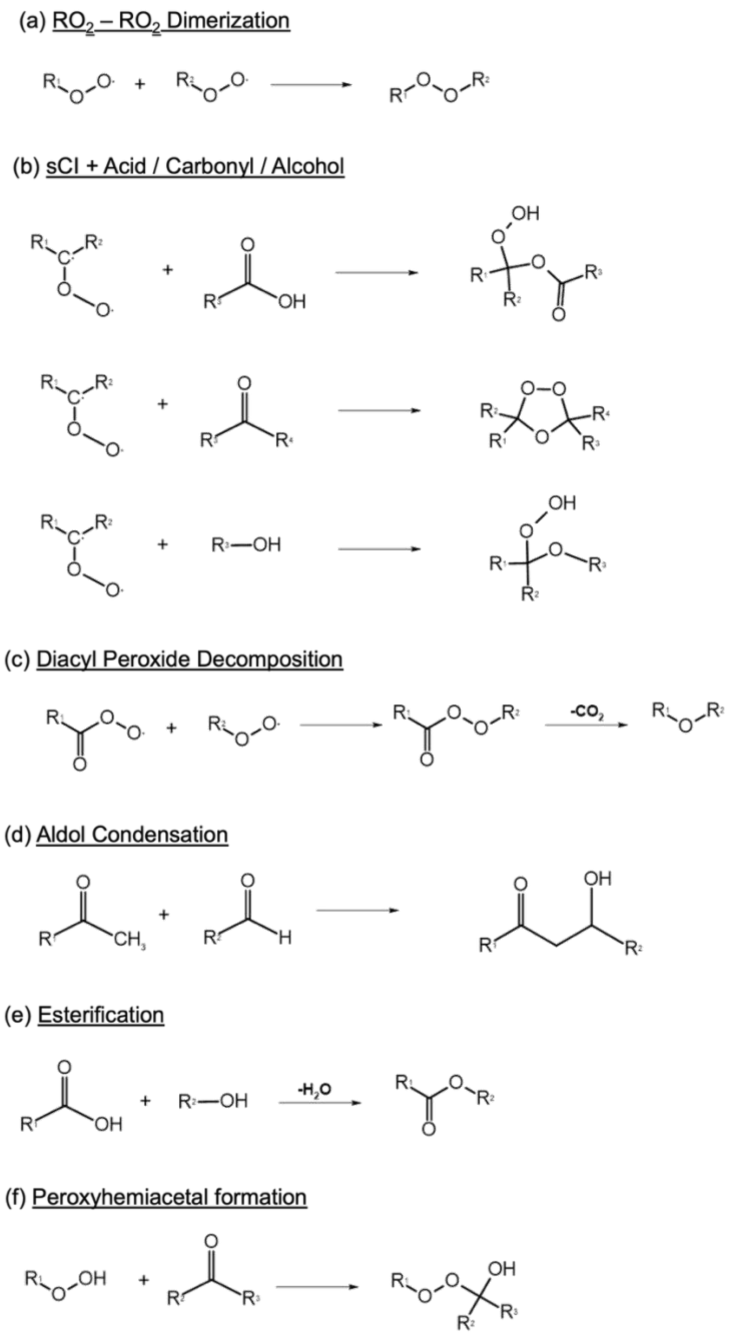

Figure S2. An overview of known reaction pathways for OOM dimers from α-pinene ozonolysis available in the literature. Gas-phase dimerization includes cross-reactions of organic peroxy radicals (RO_2)[2] (a) and sCI (stabilized Criegee intermediate) reacting with acids, carbonyls and alcohol[3] (b). Particle-phase decomposition includes diacyl peroxide decomposition[4] (c). Particle-

phase dimerizations include aldol condensation (d), esterification (e), and peroxyhemiacetal formation (f).[3]

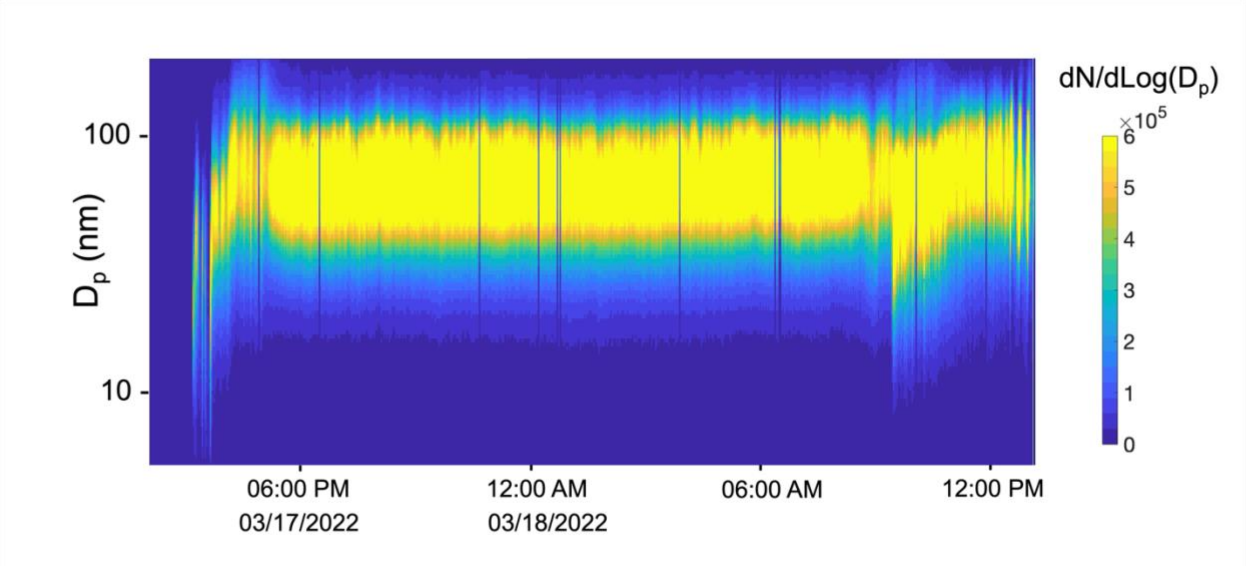

Figure S3. The averaged particle number size distribution of biogenic new articles produced from the α-pinene ozonolysis in the TANGENT.[5-7] [α-pinene] = 240 ppb, [O_3] = 1.2 ppm , Temperature of 298 K, RH < 10 %, [OH] = 1.6 ppt, and residence time = 150 s. The OH concentrations were calculated from the box model simulations, as discussed in detail in Tiszenkel and Lee[6]. The mean number size was approximately 70 nm. The mass concentration was 135 ± 23 μg m^{-3}, when assuming the density of particles as 1 g cm^{-3}.

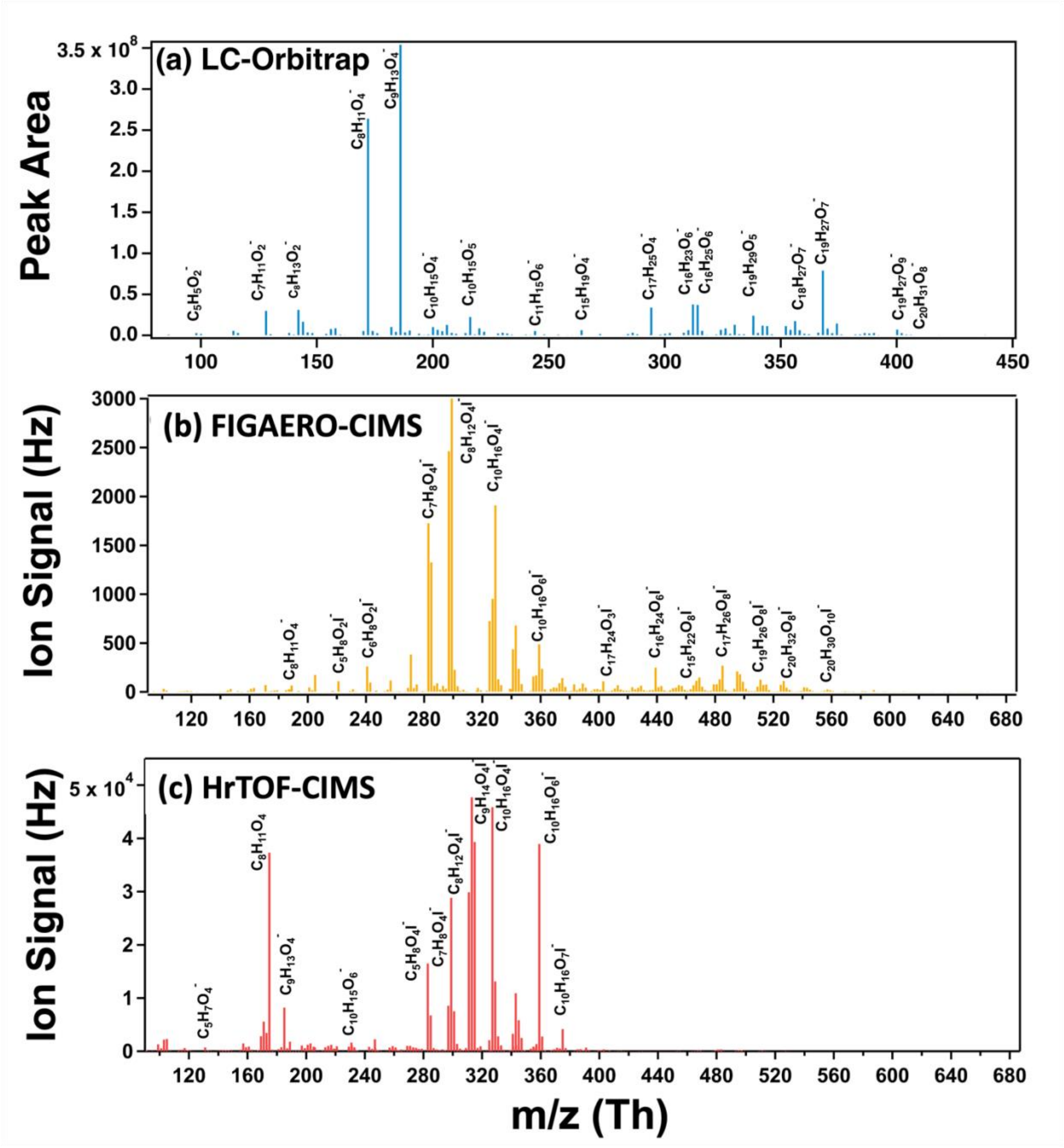

Figure S4. Linear scale mass spectra (a, b, and c) of particle-phase OOMs measured with UPLC/(-)ESI-Orbitrap MS (a), particle-phase OOMs measured with FIGAERO iodide HrTOF-CIMS (b), and gas-phase OOMs measured with iodide HrTOF-CIMS (c).

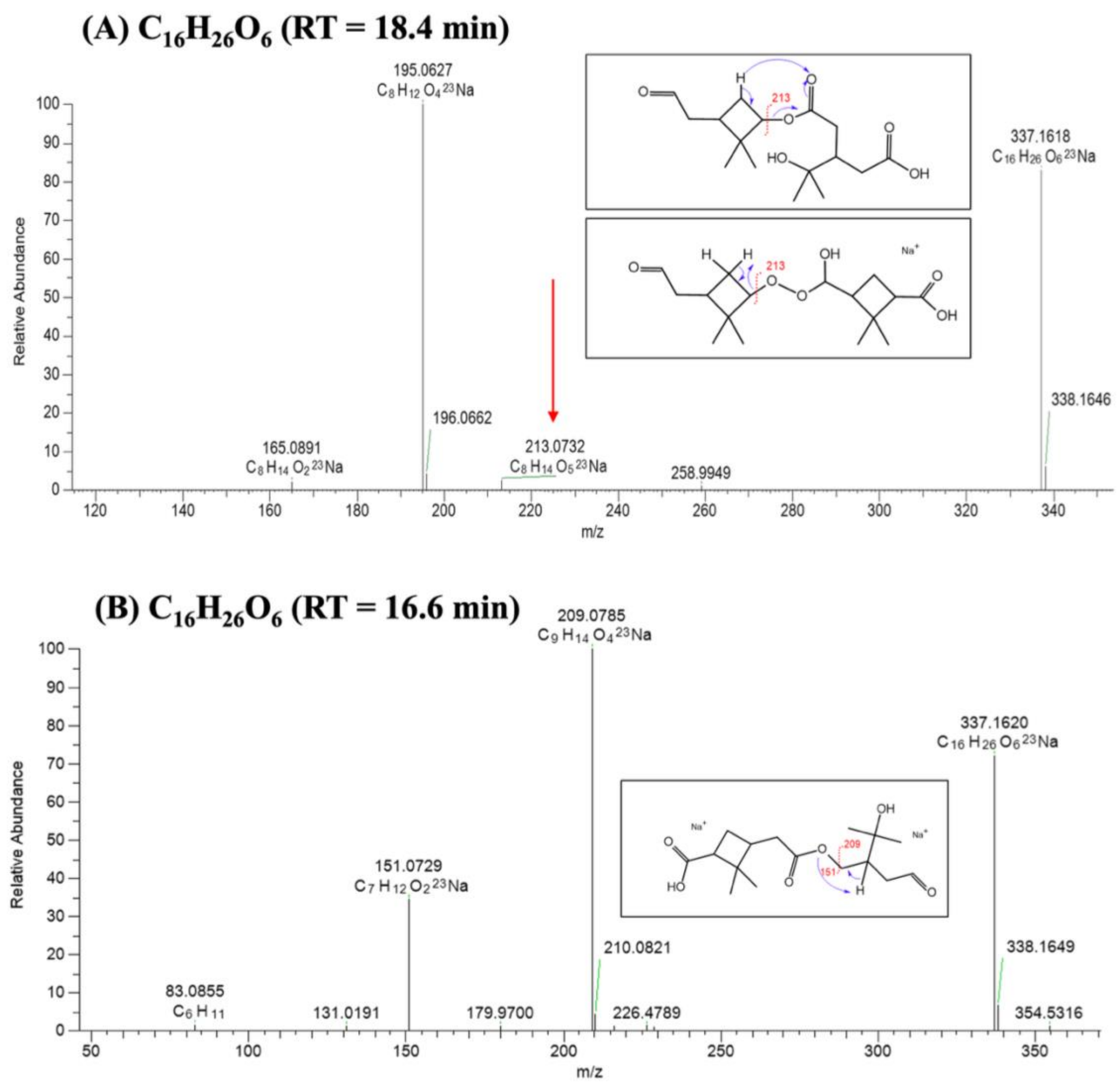

Figure S5. (a) The Na^+ adduct positive mode MS/MS spectrum of $C_{16}H_{26}O_6$ (RT = 18.4 min) isomer showing the monomeric units $C_8H_{12}O_4$ (m/z 195.0627). $C_8H_{14}O_2$ (m/z = 165.0891) and $C_8H_{14}O_5$ (m/z 213.0732) could be fragmented from the two products following the McLafferty rearrangement and remote hydrogen rearrangement, respectively, as shown in the inset. (b) The Na^+ adduct positive mode MS/MS spectrum of $C_{16}H_{26}O_6$ (RT = 16.6 min) (m/z = 337.1620) showing the two monomeric units $C_9H_{14}O_4$ (m/z 209.0785) and $C_7H_{12}O_2$ (m/z 151.0729) fragmented by remote hydrogen rearrangement as shown in inset.[8]

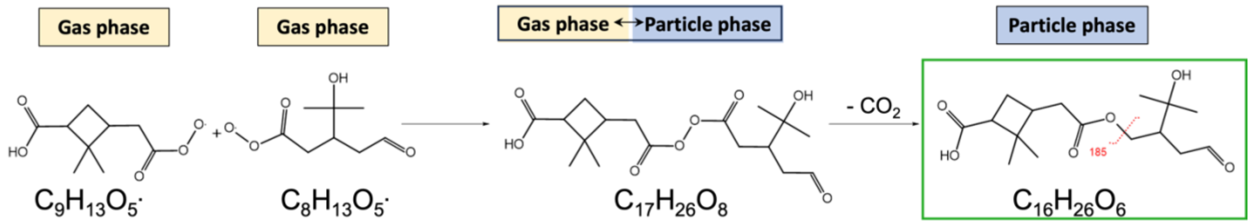

Figure S6. Possible formation mechanism of $C_{16}H_{26}O_6$ (RT = 16.6 min) (see Figure 3) via diacyl decomposition reaction, considered in our analysis but excluded as a possibility because $C_8H_{13}O_5^{\cdot}$ was not detected with CIMS.

Table S1: Organic compounds identified in this study, which match with GECKO-A simulations. The GECKO formats and their ranking during simulation (#) and modeled gas-phase concentrations are also included.

Monomer Structure	Matched Species Name in GECKO-A α-Pinene Scheme ("No match" if not found) with GECKO-A format and ranking during simulation if applicable	Modelled gas-phase concentrations (ppb)
1. $C_9H_{15}O_3$ (MW 171)<br>RO_2-1<br>MCM - C96O2<br>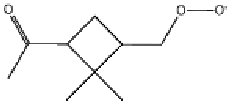	**2T9004**<br>CH3COC1HCH2CH(CH2(OO.))C1(CH3)CH3<br># 24 out of total # 90017 radicals	0.053
2. $C_{10}H_{15}O_4$ (MW 199)<br>RO_2-2<br>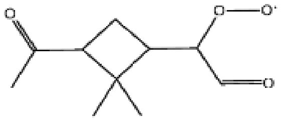	**2T0006**<br>CH3COC1HCH2CH(C1(CH3)CH3)CH(OO.)CHO<br># 5 out of total # 90017 radicals	0.71
3. $C_{10}H_{15}O_4$ (MW 199)<br>RO_2-3<br>MCM - C109O2<br>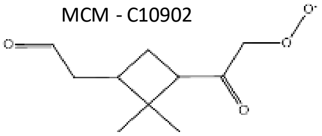	**2T0008**<br>CHOCH2C1HCH2CH(C1(CH3)CH3)COCH2(OO .)<br># 8 out of total # 90017 radicals	0.17
4. $C_{10}H_{16}O_3$ (MW 184)<br>sCI-1<br>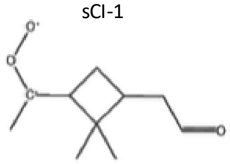	**4T0002**<br>CH3C.(ZOO.)CH(C1(CH3)CH3)CH2C1HCH2CH O<br># 1 out of total # 90017 radicals.	1.16
5. $C_{10}H_{16}O_3$ (MW 184)<br>sCI-2<br>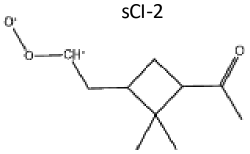	**4T0001**<br>CH3COCH(C1(CH3)CH3)CH2C1HCH2CH.(EO O.)<br># 300 out of total # 90017 radicals	2.70E-04
6. $C_9H_{14}O_3$ (MW 170)<br>Pinalic-4-acid<br>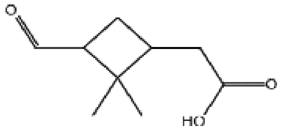	**No Match**<br>CO(OH)CH2C1HCH2CH(CHO)C1(CH3)CH3	
7. $C_9H_{14}O_3$ (MW 170)<br>Pinalic-3-acid<br>MCM - C89CO2H<br>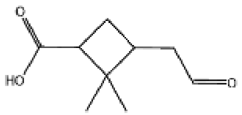	**TA9000**<br>CH3C1(CH3)CH(CO(OH))CH2C1HCH2CHO<br># 34 out of total # 121082 species	0.74

8. $C_9H_{14}O_3$ (MW 170)<br>Norpinonic acid<br>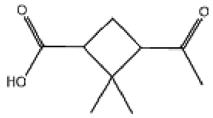	**TA9003**<br>CH3COC1HCH2CH(CO(OH))C1(CH3)CH3<br># 119 out of total # 121082 species	0.075
9. $C_{10}H_{16}O_3$ (MW 184)<br>Pinonic acid<br>MCM - PINONIC<br>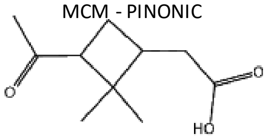	**TA0000**<br>CH3COCH(C1(CH3)CH3)CH2C1HCH2CO(OH)<br># 3 out of total # 121082 species	7.86
10. $C_{10}H_{16}O_3$ (MW 184)<br>10-Hydroxypinonaldehyde<br>MCM - C109OH<br>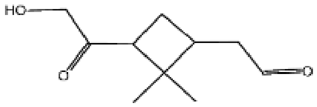	**TD0005**<br>CH3C1(CH3)CH(COCH2(OH))CH2C1HCH2CHO<br># 17 out of total # 121082<br>species	2.93
11. $C_9H_{14}O_2$ (MW 154)<br>Norpinonaldehyde<br>MCM - NORPINAL<br>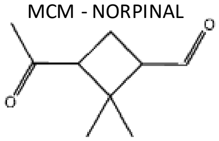	**TD9000**<br>CH3COC1HCH2CH(CHO)C1(CH3)CH3<br># 5 out of total # 121082 species	7.68
12. $C_{10}H_{16}O_2$ (MW 168)<br>Pinonaldehyde<br>MCM - PINAL<br>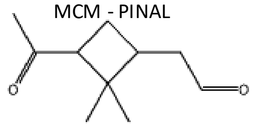	**TD0000**<br>CH3COCH(C1(CH3)CH3)CH2C1HCH2CHO<br># 1 out of total # 121082 species	14.41
13. $C_9H_{14}O_4$ (MW 186)<br>Cis-3-peroxypinalic acid<br>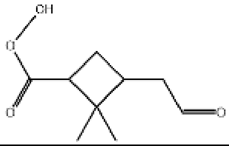	**TG9000**<br>CH3CH(OH)CH(C1(CH3)CH3)CH2C1HCH2CHO	0.05
14. $C_{10}H_{18}O_2$ (MW 170)<br>α-Pinanediol<br>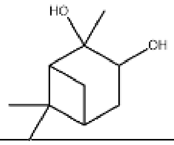	**TT000D**<br>C12HCH2CH(C1(CH3)CH3)CH2CH(OH)C2(OH)CH3	3
15. $C_9H_{13}O_5$ (MW 201)<br>MCM - C811CO3<br>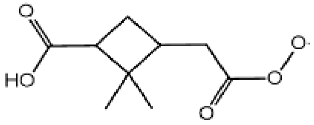	**3T9001**<br>CH3C1(CH3)CH(CO(OH))CH2C1HCH2CO(OO.)	0.021

82

16. $C_8H_{13}O_5$ (MW 189)<br>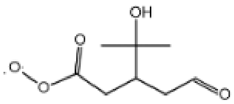	**3D8000**<br>CH3C(OH)(CH3)CH(CH2CHO)CH2CO(OO.)	1.92E-04
17. $C_9H_{13}O_4$ (MW 185)<br>MCM - C89CO3<br>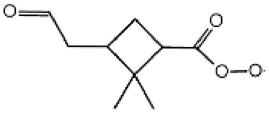	**3T9000**<br>CHOCH2C1HCH2CH(CO(OO.))C1(CH3)CH3<br># 15 out of total # 90017 radicals	0.029
18. $C_8H_{13}O_6$ (MW 205)<br>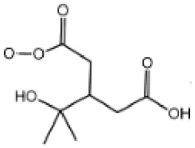	**3A8004**<br>CH3C(OH)(CH3)CH(CH2CO(OH))CH2CO(OO.)<br># 2.040 out of total # 90017 radicals	1.34E-06
19. $C_8H_{14}O_3$ (MW 158)<br>MCM - C89OOH<br>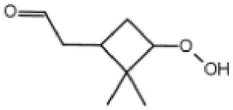	**TH8000**<br>CH3C1(CH3)CH(OOH)CH2C1HCH2CHO<br># 39 out of total # 121082 species	0.5
20. $C_8H_{12}O_3$ (MW 156)<br>Norpinalic acid<br>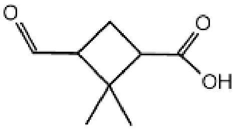	**TA8002**<br>CO(OH)C1HCH2CH(CHO)C1(CH3)CH3<br># 8 out of total # 121082 species	4.5

83

Table S2. Monomer building blocks for the dimer structures for $C_{19}H_{30}O_5$ formed from possible reactions in the α-pinene ozonolysis system. The structures inside the green boxes match with the fragments observed for $C_{19}H_{30}O_5$ (RT = 15.3 min). Gray cells indicate no possible reactions between any possible building blocks.

Monomer Building blocks	1. $C_9H_{15}O_3$ (MW 171) RO$_2$-1 MCM - C96O2 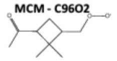	2. $C_{10}H_{16}O_3$ (MW 184) sCI-1 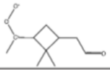	3. $C_{10}H_{16}O_3$ (MW 184) sCI-2 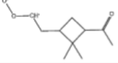	4. $C_9H_{14}O_3$ (MW 170) Pinalic-4-acid 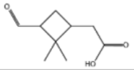	5. $C_9H_{14}O_3$ (MW 170) Pinalic-3-acid MCM - C89CO2H 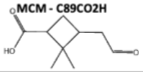	6. $C_9H_{14}O_3$ (MW 170) Norpinonic acid 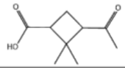	7. $C_{10}H_{16}O_3$ (MW 184) Pinonic acid MCM - PINONIC 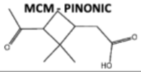	8. $C_{10}H_{16}O_3$ (MW 184) 10-Hydroxypinonaldehyde MCM - C109OH 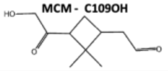
A. $C_{10}H_{15}O_4$ (MW 199) RO$_2$-2 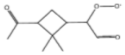	1A. $C_{19}H_{30}O_5$ (MW 338) RO$_2$ - RO$_2$ 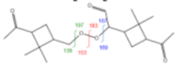							
B. $C_{10}H_{15}O_4$ (MW 199) RO$_2$-3 MCM - C109O2 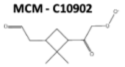	1B. $C_{19}H_{30}O_5$ (MW 338) RO$_2$ - RO$_2$ 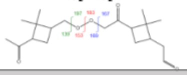							
C. $C_{10}H_{16}O_3$ (MW 184) sCI-1 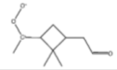		2C. $C_{20}H_{34}O_4$ (MW 338) sCI + sCI = ROOR	3C. $C_{20}H_{34}O_4$ (MW 338) sCI + sCI = ROOR	4C. $C_{19}H_{30}O_6$ (MW 354) sCI + acid = α-AAHP	5C. $C_{19}H_{30}O_6$ (MW 354) sCI + acid = α-AAHP	6C. $C_{19}H_{30}O_6$ (MW 354) sCI + acid = α-AAHP	7C. $C_{20}H_{32}O_6$ (MW 368) sCI + acid = α-AAHP	8C. $C_{20}H_{32}O_6$ (MW 368) sCI + Aldeheyde = SOZ
D. $C_9H_{14}O_2$ (MW 154) Norpinonaldehyde MCM - NORPINAL 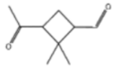		2D.1. $C_{19}H_{30}O_5$ (MW 338) sCI + Aldeheyde = SOZ 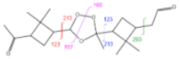 2D.2. $C_{19}H_{30}O_5$ (MW 338) sCI + Ketone = SOZ 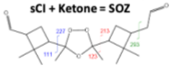	3D.1. $C_{19}H_{30}O_5$ (MW 338) sCI + Aldeheyde = SOZ 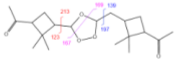 3D.2. $C_{19}H_{30}O_5$ (MW 338) sCI + Ketone = SOZ 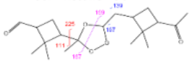	4D. $C_{18}H_{28}O_5$ (MW 324) Aldol condensation	5D. $C_{18}H_{28}O_5$ (MW 324) Aldol condensation	6D. $C_{18}H_{28}O_5$ (MW 324) Aldol condensation	7D.1. $C_{19}H_{30}O_5$ (MW 338) Aldol condensation 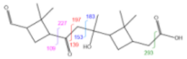 7D.2. $C_{19}H_{30}O_5$ (MW 338) Aldol condensation 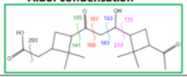	8D.1. $C_{19}H_{30}O_5$ (MW 338) Aldol condensation 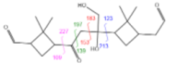 8D.2. $C_{19}H_{30}O_5$ (MW 338) Aldol condensation 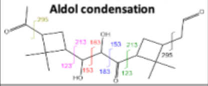
E. $C_{10}H_{16}O_2$ (MW 168) Pinonaldehyde MCM - PINAL 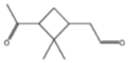		2E. $C_{20}H_{32}O_5$ (MW 352) sCI + Aldeheyde = SOZ	3E. $C_{20}H_{32}O_5$ (MW 352) sCI + Aldeheyde = SOZ	4E.1. $C_{19}H_{30}O_5$ (MW 338) Aldol condensation 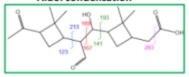 4E.2. $C_{19}H_{30}O_5$ (MW 338) Aldol condensation 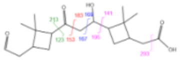	5E.1. $C_{19}H_{30}O_5$ (MW 338) Aldol condensation 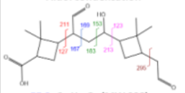 5E.2. $C_{19}H_{30}O_5$ (MW 338) Aldol condensation 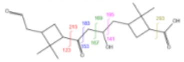	6E.1. $C_{19}H_{30}O_5$ (MW 338) Aldol condensation 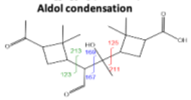 6E.1. $C_{19}H_{30}O_5$ (MW 338) Aldol condensation 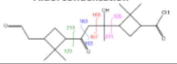	7E. $C_{20}H_{32}O_5$ (MW 352) Aldol condensation	8E. $C_{20}H_{32}O_5$ (MW 352) Aldol condensation

Table S3. The top 50% OOMs in gas- and particle-phase detected with FIGAERO HrTOF-CIMS, and the particle-phase OOMs detected in UPLC/(-)ESI-Orbitrap mass spectrometer with S/N > 3. Check signs indicate detection.

Compounds	HrTOF-CIMS (Gas)	FIGAERO -CIMS (Particle)	UPLC-Orbitrap (Particle)
C5H10O			✓
C5H10O3	✓	✓	
C5H10O4		✓	
C5H10O5	✓	✓	
C5H10O6	✓	✓	
C5H10O7	✓	✓	
C5H10O8	✓		
C5H4O3	✓		
C5H4O5	✓	✓	
C5H6O2	✓		✓
C5H6O3	✓		
C5H6O4	✓	✓	
C5H6O5	✓	✓	
C5H6O6	✓	✓	
C5H6O7	✓		
C5H7O5	✓		
C5H7O7	✓		
C5H8O2	✓	✓	✓
C5H8O3	✓	✓	✓
C5H8O4	✓	✓	
C5H8O5	✓	✓	✓
C5H8O6	✓		
C5H8O7	✓	✓	
C5H8O8	✓		
C6H10O			✓
C6H10O2	✓		✓
C6H10O3			✓
C6H10O4	✓	✓	✓
C6H10O5	✓	✓	
C6H10O6	✓	✓	
C6H10O7	✓		
C6H10O8	✓		
C6H11O4	✓		
C6H12O4	✓		
C6H12O5	✓		
C6H12O6	✓		
C6H12O8	✓	✓	
C6H14O8	✓	✓	
C6H15O6	✓		
C6H6O4	✓	✓	
C6H6O6		✓	
C6H7O2	✓		
C6H8O2	✓		
C6H8O3	✓	✓	
C6H8O4	✓	✓	✓
C6H8O5	✓	✓	
C6H8O6	✓	✓	
C6H8O7	✓	✓	
C7H10O3	✓	✓	✓
C7H10O4	✓	✓	✓
C7H10O5	✓	✓	✓
C7H10O6	✓	✓	✓
C7H10O7	✓	✓	
C7H10O8	✓		
C7H10O9	✓		
C7H11O5		✓	
C7H11O6	✓		
C7H12O2			✓
C7H12O3	✓	✓	
C7H12O4	✓	✓	✓
C7H12O5	✓	✓	✓
C7H12O6	✓	✓	
C7H12O7	✓	✓	
C7H12O8	✓		

C7H12O9	✔		
C7H13O5	✔	✔	
C7H13O8	✔		
C7H13O9		✔	
C7H14O2			✔
C7H14O3			✔
C7H14O4	✔	✔	✔
C7H14O5	✔	✔	
C7H14O6	✔	✔	
C7H14O7	✔	✔	
C7H14O8	✔	✔	
C7H16O7	✔	✔	
C7H5O3	✔		
C7H8O2	✔		
C7H8O3	✔		✔
C7H8O4	✔	✔	
C8H10O3	✔	✔	✔
C8H10O4	✔	✔	
C8H10O5	✔		✔
C8H11O6	✔		
C8H12O10	✔		
C8H12O2	✔		
C8H12O3	✔		✔
C8H12O4	✔	✔	✔
C8H12O5	✔	✔	✔
C8H12O6	✔	✔	✔
C8H12O7	✔	✔	
C8H12O8	✔	✔	
C8H12O9	✔		
C8H13O7	✔		
C8H14O2	✔		✔
C8H14O3	✔		✔
C8H14O4		✔	✔
C8H14O5	✔	✔	✔
C8H14O6	✔	✔	✔
C8H14O7	✔	✔	
C8H14O8	✔		
C8H14O9	✔	✔	✔
C8H16O10	✔	✔	

C8H16O3			✔
C8H16O8	✔		
C8H16O9	✔		
C8H18O10	✔	✔	
C8H18O9	✔	✔	
C8H8O2			✔
C8H8O3	✔	✔	
C8H8O4	✔	✔	
C8H8O9	✔		
C9H10O10	✔	✔	
C9H10O11	✔	✔	✔
C9H10O13			✔
C9H10O3	✔		
C9H10O5	✔		
C9H10O7	✔		
C9H10O8	✔		
C9H10O9		✔	
C9H11O3	✔		
C9H11O6	✔		
C9H11O8	✔		
C9H11O9	✔		
C9H12O3	✔	✔	
C9H12O4	✔		✔
C9H12O5	✔		
C9H12O6	✔	✔	✔
C9H12O8	✔	✔	
C9H12O9	✔		
C9H13O5	✔		
C9H13O6	✔		
C9H13O7	✔		
C9H14O			✔
C9H14O2	✔		✔
C9H14O3	✔	✔	✔
C9H14O4	✔	✔	✔
C9H14O5	✔	✔	✔
C9H14O6	✔	✔	✔
C9H14O7	✔	✔	
C9H14O8	✔		
C9H14O9	✔		

C9H15O4	✓	✓	
C9H15O6	✓		
C9H15O7	✓		
C9H15O8	✓		
C9H16O			✓
C9H16O11	✓		
C9H16O2			✓
C9H16O3			✓
C9H16O5	✓	✓	✓
C9H16O6	✓	✓	✓
C9H16O7	✓	✓	
C9H16O8	✓	✓	
C9H18O10	✓		
C9H18O11	✓	✓	
C9H18O2	✓		
C9H18O3			✓
C9H20O10	✓	✓	
C9H20O5	✓		
C9H20O8	✓	✓	
C9H6O5	✓		
C10H10O4	✓	✓	
C10H10O5			✓
C10H11O5	✓		
C10H12O11	✓	✓	
C10H12O4	✓	✓	
C10H12O5	✓		
C10H12O6	✓	✓	
C10H12O7	✓	✓	
C10H12O8	✓		
C10H13O5	✓	✓	
C10H13O6	✓		
C10H13O7	✓		
C10H14O		✓	
C10H14O10		✓	
C10H14O12	✓	✓	
C10H14O2	✓	✓	
C10H14O3	✓	✓	✓
C10H14O4	✓	✓	✓
C10H14O5	✓	✓	✓

C10H14O6	✓	✓	✓
C10H14O7	✓	✓	
C10H14O8	✓	✓	
C10H14O9	✓	✓	
C10H15O4	✓		
C10H15O6	✓		
C10H15O7	✓		
C10H15O8	✓		
C10H16O10	✓		
C10H16O2	✓		
C10H16O3	✓	✓	✓
C10H16O4	✓	✓	✓
C10H16O5	✓	✓	✓
C10H16O6	✓	✓	✓
C10H16O7	✓	✓	✓
C10H16O8	✓	✓	
C10H16O9	✓	✓	
C10H17O6	✓		
C10H17O7	✓		
C10H17O8	✓		
C10H18O10	✓	✓	✓
C10H18O4			✓
C10H18O5	✓	✓	
C10H18O6	✓	✓	✓
C10H18O7	✓		
C10H18O8	✓	✓	
C10H18O9	✓		
C10H20O10	✓	✓	
C10H20O3			✓
C10H20O6	✓	✓	
C10H20O8	✓	✓	
C10H20O9	✓	✓	
C10H22O4	✓	✓	
C10H22O6	✓		
C10H22O8	✓	✓	
C11H10O2	✓		
C11H12O10	✓		
C11H12O6	✓	✓	
C11H12O7	✓	✓	

C11H14O10	✓	✓	
C11H14O2	✓	✓	
C11H14O3	✓	✓	✓
C11H14O6	✓	✓	
C11H14O7	✓	✓	
C11H14O9	✓		
C11H16O	✓		
C11H16O6	✓	✓	✓
C11H16O7	✓	✓	
C11H16O8	✓	✓	
C11H16O9	✓	✓	
C11H17O4		✓	
C11H17O6	✓		
C11H17O7	✓		
C11H17O8	✓		
C11H18O10	✓		
C11H18O4			✓
C11H18O5	✓	✓	✓
C11H18O6		✓	
C11H18O7	✓	✓	✓
C11H19O3	✓		
C11H19O7	✓		
C11H19O8	✓		
C11H20O10	✓		
C11H20O11	✓	✓	
C11H20O4			✓
C11H24O10	✓		
C11H24O3	✓	✓	
C11H24O6	✓	✓	
C11H24O7		✓	
C12H10O6	✓		
C12H10O8	✓	✓	
C12H12O5		✓	
C12H12O8	✓		
C12H14O4			✓
C12H14O7	✓	✓	
C12H16O10	✓		
C12H16O11	✓	✓	
C12H16O2	✓		

C12H16O3			✓
C12H16O6	✓		
C12H16O7	✓	✓	
C12H16O8	✓		
C12H17O7	✓		
C12H18O5			✓
C12H18O6	✓		✓
C12H18O7	✓	✓	
C12H18O8	✓	✓	
C12H18O9	✓		
C12H19O7	✓		
C12H20O2			✓
C12H20O4			✓
C12H20O5	✓	✓	✓
C12H20O6			✓
C12H20O7	✓		
C12H20O8	✓	✓	
C12H22O11			✓
C12H22O3			✓
C12H22O4			✓
C12H24O5	✓	✓	
C12H24O6	✓	✓	
C12H26O6	✓	✓	
C12H8O12	✓	✓	
C13H14O5	✓	✓	
C13H16O6	✓		
C13H16O9	✓	✓	
C13H18O3	✓		
C13H18O9	✓	✓	
C13H20O2			✓
C13H20O4			✓
C13H20O5	✓		
C13H20O6	✓	✓	✓
C13H20O7	✓	✓	✓
C13H20O8	✓		
C13H20O9		✓	
C13H22O2			✓
C13H22O6		✓	
C13H22O7	✓	✓	

C13H22O8	✔	✔	
C13H24O14	✔	✔	
C13H24O5	✔	✔	
C13H26O16			✔
C13H28O8		✔	
C14H16O6	✔		
C14H18O4	✔	✔	
C14H20O2	✔	✔	
C14H20O6			✔
C14H20O7	✔	✔	✔
C14H20O8		✔	✔
C14H22O12	✔	✔	
C14H22O2			✔
C14H22O4			✔
C14H22O5	✔		
C14H22O6	✔	✔	✔
C14H22O8	✔	✔	
C14H24O10	✔	✔	
C14H24O11	✔		
C14H24O6	✔	✔	
C14H24O7	✔	✔	
C14H26O13	✔	✔	
C14H26O6		✔	
C14H26O8		✔	
C14H28O16			✔
C14H28O6	✔	✔	
C14H28O9	✔		
C14H30O5	✔	✔	
C15H17O5	✔		
C15H18O2		✔	
C15H18O3	✔	✔	
C15H18O5	✔	✔	
C15H20O10	✔	✔	
C15H20O4			✔
C15H20O5		✔	
C15H20O9	✔	✔	
C15H22O10		✔	
C15H22O11	✔	✔	
C15H22O6	✔	✔	✔

C15H22O8	✔	✔	✔
C15H22O9	✔	✔	
C15H24O10	✔	✔	
C15H24O11	✔		
C15H24O5	✔	✔	✔
C15H24O6	✔	✔	✔
C15H24O7		✔	✔
C15H24O8	✔	✔	✔
C15H24O9		✔	
C15H25O9	✔		
C15H26O10	✔	✔	
C15H26O13	✔	✔	
C15H26O5			✔
C15H26O6	✔	✔	✔
C15H26O7			✔
C15H26O8			✔
C15H27O5		✔	
C15H28O12	✔	✔	
C15H28O13	✔	✔	
C15H30O4	✔	✔	
C15H30O9	✔		
C15H31O12	✔		
C15H31O9	✔		
C15H32O12		✔	
C15H32O5	✔		
C15H32O9	✔		
C16H16O6	✔	✔	
C16H18O2	✔	✔	
C16H18O3	✔	✔	
C16H18O7		✔	
C16H20O11	✔	✔	
C16H20O5	✔	✔	
C16H20O6		✔	
C16H20O7	✔	✔	
C16H20O8	✔	✔	
C16H20O9	✔	✔	
C16H22O10	✔	✔	
C16H22O11	✔	✔	
C16H22O12		✔	

C16H22O4		✓	
C16H22O5	✓	✓	
C16H22O6		✓	
C16H22O7	✓	✓	✓
C16H22O8	✓	✓	
C16H22O9	✓	✓	
C16H24O11	✓	✓	
C16H24O5		✓	
C16H24O6	✓	✓	✓
C16H24O7	✓	✓	✓
C16H24O8	✓	✓	✓
C16H26O10		✓	
C16H26O4			✓
C16H26O5		✓	✓
C16H26O6		✓	✓
C16H26O7	✓	✓	✓
C16H26O8	✓	✓	✓
C16H26O9		✓	✓
C16H28O7		✓	
C16H30O3	✓	✓	
C16H31O12	✓		
C16H32O8	✓	✓	
C16H34O4	✓	✓	
C16H34O5	✓	✓	
C17H14O12	✓	✓	
C17H14O6	✓	✓	
C17H14O7	✓		
C17H18O2	✓	✓	
C17H18O5	✓	✓	
C17H18O7	✓	✓	
C17H20O7		✓	
C17H20O9		✓	
C17H22O10	✓	✓	
C17H22O7	✓	✓	
C17H22O8	✓		
C17H22O9	✓	✓	
C17H24O12		✓	
C17H24O3	✓	✓	
C17H24O6	✓	✓	✓

C17H24O7	✓	✓	
C17H24O8	✓	✓	✓
C17H24O9	✓	✓	
C17H25O5		✓	
C17H25O9	✓		
C17H26O4			✓
C17H26O5	✓	✓	✓
C17H26O6	✓	✓	✓
C17H26O7	✓	✓	✓
C17H26O8	✓	✓	✓
C17H26O9	✓	✓	✓
C17H27O2	✓		
C17H28O10			✓
C17H28O11		✓	
C17H28O12		✓	
C17H28O3	✓	✓	
C17H28O4		✓	
C17H28O5		✓	✓
C17H28O6	✓	✓	
C17H28O7	✓	✓	✓
C17H28O8		✓	✓
C17H28O9		✓	✓
C17H30O4	✓	✓	
C17H30O5	✓	✓	
C17H32O4	✓	✓	
C17H32O5	✓	✓	
C17H32O6	✓	✓	
C17H36O4	✓		
C17H36O5	✓		
C18H14O8			✓
C18H18O5	✓	✓	
C18H20O6	✓	✓	
C18H22O13	✓	✓	
C18H22O7	✓	✓	
C18H24O10	✓	✓	
C18H24O14		✓	
C18H24O2	✓	✓	
C18H24O5		✓	
C18H24O6	✓	✓	

C18H24O8	✓	✓	
C18H24O9	✓	✓	
C18H26O5	✓	✓	✓
C18H26O6	✓	✓	✓
C18H26O7	✓	✓	✓
C18H26O8	✓	✓	✓
C18H26O9	✓	✓	✓
C18H28O10			✓
C18H28O11		✓	
C18H28O4	✓	✓	✓
C18H28O5	✓	✓	✓
C18H28O6	✓	✓	✓
C18H28O7	✓	✓	✓
C18H28O8	✓	✓	✓
C18H28O9	✓	✓	✓
C18H30O10			✓
C18H30O15		✓	
C18H30O3	✓	✓	
C18H30O4		✓	✓
C18H30O5	✓	✓	✓
C18H30O6	✓	✓	✓
C18H30O7	✓	✓	✓
C18H30O8		✓	✓
C18H30O9		✓	✓
C18H32O12		✓	
C18H32O18	✓	✓	
C18H32O6	✓	✓	
C18H32O7	✓	✓	
C18H32O8		✓	
C18H32O9		✓	
C18H34O12		✓	
C18H34O5	✓	✓	
C18H34O6	✓	✓	
C18H34O7		✓	
C18H34O8		✓	
C18H36O12		✓	
C18H36O2	✓	✓	
C18H36O9	✓	✓	
C19H17O9		✓	

C19H20O17		✓	
C19H22O4	✓	✓	
C19H22O6		✓	
C19H24O10		✓	
C19H26O4	✓	✓	
C19H26O5	✓	✓	
C19H26O6	✓	✓	
C19H26O7	✓		
C19H26O8	✓	✓	
C19H26O9	✓	✓	
C19H28O11	✓	✓	
C19H28O4		✓	
C19H28O5	✓	✓	
C19H28O6	✓	✓	✓
C19H28O7	✓	✓	✓
C19H28O8	✓	✓	✓
C19H28O9	✓	✓	✓
C19H30O10	✓	✓	✓
C19H30O11	✓	✓	
C19H30O12		✓	
C19H30O13		✓	
C19H30O15		✓	
C19H30O4	✓	✓	
C19H30O5	✓	✓	✓
C19H30O6	✓	✓	✓
C19H30O7	✓	✓	✓
C19H30O8	✓	✓	✓
C19H30O9	✓	✓	✓
C19H32O10	✓	✓	
C19H32O11		✓	
C19H32O12		✓	
C19H32O13		✓	
C19H32O14		✓	
C19H32O15		✓	
C19H32O6	✓	✓	
C19H32O7			✓
C19H32O8		✓	
C19H32O9	✓		
C19H34O7	✓	✓	

$C_{19}H_{34}O_{9}$		✓		
$C_{19}H_{36}O_{12}$		✓		
$C_{19}H_{38}O_{10}$	✓	✓		
$C_{19}H_{42}O_{6}$	✓	✓		
$C_{20}H_{18}O_{12}$			✓	
$C_{20}H_{18}O_{9}$			✓	
$C_{20}H_{22}O_{10}$	✓			
$C_{20}H_{24}O_{6}$	✓	✓		
$C_{20}H_{24}O_{7}$		✓		
$C_{20}H_{26}O_{10}$		✓		
$C_{20}H_{26}O_{5}$		✓		
$C_{20}H_{26}O_{6}$		✓		
$C_{20}H_{28}O_{10}$	✓	✓		
$C_{20}H_{28}O_{11}$		✓		
$C_{20}H_{28}O_{12}$	✓	✓		
$C_{20}H_{28}O_{5}$	✓	✓		
$C_{20}H_{28}O_{6}$	✓	✓		
$C_{20}H_{28}O_{7}$	✓	✓		4
$C_{20}H_{28}O_{8}$		✓		
$C_{20}H_{30}O_{10}$	✓	✓		
$C_{20}H_{30}O_{11}$		✓		
$C_{20}H_{30}O_{12}$		✓		
$C_{20}H_{30}O_{13}$	✓			
$C_{20}H_{30}O_{14}$		✓		
$C_{20}H_{30}O_{15}$		✓		
$C_{20}H_{30}O_{16}$		✓		
$C_{20}H_{30}O_{5}$	✓	✓		
$C_{20}H_{30}O_{6}$	✓	✓	✓	
$C_{20}H_{30}O_{7}$	✓	✓	✓	
$C_{20}H_{30}O_{8}$	✓	✓		
$C_{20}H_{30}O_{9}$	✓	✓		
$C_{20}H_{32}O_{10}$	✓	✓		
$C_{20}H_{32}O_{11}$		✓		
$C_{20}H_{32}O_{13}$		✓		
$C_{20}H_{32}O_{14}$		✓		
$C_{20}H_{32}O_{15}$		✓		
$C_{20}H_{32}O_{16}$		✓		
$C_{20}H_{32}O_{4}$	✓	✓		
$C_{20}H_{32}O_{6}$	✓	✓	✓	

$C_{20}H_{32}O_{7}$	✓	✓	✓
$C_{20}H_{32}O_{8}$	✓	✓	✓
$C_{20}H_{32}O_{9}$	✓	✓	
$C_{20}H_{34}O_{13}$		✓	
$C_{20}H_{34}O_{3}$		✓	
$C_{20}H_{34}O_{5}$	✓	✓	
$C_{20}H_{34}O_{6}$	✓	✓	
$C_{20}H_{34}O_{7}$	✓	✓	
$C_{20}H_{34}O_{8}$	✓	✓	
$C_{20}H_{38}O_{11}$		✓	
$C_{20}H_{38}O_{3}$		✓	
$C_{20}H_{38}O_{5}$	✓	✓	
$C_{20}H_{38}O_{9}$		✓	
$C_{20}H_{40}O_{10}$		✓	
$C_{20}H_{40}O_{9}$		✓	
$C_{20}H_{42}O_{10}$		✓	
$C_{20}H_{42}O_{11}$		✓	

Table S4. Isomers of OOMs identified by UPLC/(-)ESI-Orbitrap MS, corresponding *m/z* values for [M-H]$^-$ ions, retention times (RT), relative abundance with respect to all the compounds listed in this table, and major MS/MS fragments identified in the negative ion mode.

Compounds	*m/z* [M-H]$^-$	RT (min)	Signal fraction (%)	MS/MS fragments – m/z of [M-H]$^-$ fragment ions and their corresponding ionic formulas [$C_xH_yO_z$]$^-$ given in parentheses
$C_5H_8O_5$-1	147.0298	1.35	0.14	85.0294 ($C_4H_5O_2$), 73.0294 ($C_3H_5O_2$), 59.0138 ($C_2H_3O_2$), 43.0190 (C_2H_3O)
$C_5H_8O_5$-2		1.62	0.19	
$C_7H_{10}O_5$-1	173.0454	2.37	0.33	
$C_7H_{10}O_5$-2		2.22	0.25	
$C_7H_{10}O_6$-1	189.0404	1.34	0.04	
$C_7H_{10}O_6$-2		1.58	0.08	
$C_7H_{12}O_4$	159.0661	9.24	0.05	
$C_7H_{12}O_5$-1	175.0610	2.75	0.18	
$C_7H_{12}O_5$-2		4.9	0.06	
$C_7H_{12}O_5$-3		4.73	0.04	
$C_7H_{12}O_5$-4		1.27	0.01	
$C_8H_{12}O_4$-1	171.0663	5.59	14.00	171.0661 ($C_8H_{11}O_4$)
$C_8H_{12}O_4$-2		6.99	0.10	
$C_8H_{12}O_4$-3		8.05	1.96	
$C_8H_{12}O_5$-1	187.0611	5.56	0.12	187.0616 ($C_8H_{11}O_5$), 143.0715 ($C_7H_{11}O_3$), 125.0976 ($C_8H_{13}O$), 87.0451 ($C_4H_7O_2$)

$C_8H_{12}O_5$-2		4.67	0.08	187.0616 ($C_8H_{11}O_5$), 169.0507 ($C_8H_9O_4$), 125.0976 ($C_8H_{13}O$), 97.0661 (C_6H_9O), 57.0347 (C_3H_5O)
$C_8H_{12}O_5$-3		8.34	0.05	
$C_8H_{12}O_5$-4		2.33	0.24	
$C_8H_{12}O_5$-5		5.77	0.06	
$C_8H_{12}O_5$-6		8.78	0.03	
$C_8H_{12}O_5$-7		2.55	0.12	
$C_8H_{12}O_6$-1	203.0560	5.86	0.25	185.0455 ($C_8H_9O_5$), 115.0764 ($C_6H_{11}O_2$), 97.06660 (C_6H_9O), 87.0452 ($C_4H_7O_2$), 71.0138 ($C_3H_3O_2$), 59.0138 ($C_2H_3O_2$)
$C_8H_{12}O_6$-2		5.75	0.12	185.0455 ($C_8H_9O_5$), 141.0559 ($C_7H_9O_3$), 97.06660 (C_6H_9O), 71.0138 ($C_3H_3O_2$)
$C_8H_{14}O_4$-1	173.9547	5.1	0.16	
$C_8H_{14}O_4$-2		10.42	0.12	
$C_8H_{14}O_5$-1	189.0768	3.86	0.21	171.0661 ($C_8H_{11}O_4$), 145.0869 ($C_7H_{13}O_3$), 127.0765 ($C_7H_{11}O_2$), 101.0972 ($C_6H_{13}O$), 85.0659 (C_5H_9O), 57.0347 (C_3H_5O)
$C_8H_{14}O_5$-2		10.71	0.46	
$C_8H_{14}O_5$-3		4.1	0.36	
$C_8H_{14}O_5$-4		5.77	0.19	
$C_8H_{14}O_5$-5		6.23	0.09	

$C_8H_{14}O_6$-1	205.0717	5.43	0.82	87.0090 ($C_3H_3O_3$), 85.0295 ($C_4H_5O_2$), 59.0138 ($C_2H_3O_2$), 57.0344 (C_3H_5O)
$C_8H_{14}O_6$-2		5.31	0.32	173.0448 ($C_7H_9O_5$), 111.0088 ($C_5H_3O_3$), 85.0295 ($C_4H_5O_2$), 59.0138 ($C_2H_3O_2$), 57.0344 (C_3H_5O)
$C_8H_{14}O_6$-3		1.58	0.04	131.0716 ($C_6H_{11}O_3$), 111.0088 ($C_5H_3O_3$), 99.0451 ($C_5H_7O_2$), 73.0297 ($C_3H_5O_2$), 57.0344 (C_3H_5O)
$C_8H_{14}O_9$	253.0692	9.3	0.06	
$C_9H_{14}O_4$-1	185.0815	9.28	31.46	167.0715 ($C_9H_{11}O_3$), 141.0922 ($C_8H_{13}O_2$), 123.0815 ($C_8H_{11}O$), 99.0451 ($C_5H_7O_2$), 71.0138 ($C_3H_3O_2$), 57.0347 (C_3H_5O)
$C_9H_{14}O_4$-2		11.06	0.11	
$C_9H_{14}O_5$-1	201.0768	4.91	0.33	183.0659 ($C_9H_{11}O_4$), 143.0349 ($C_6H_7O_4$), 139.0764 ($C_8H_{11}O_2$), 125.0244 ($C_6H_5O_3$), 111.0813 ($C_7H_{11}O$), 99.0450 ($C_5H_7O_2$), 81.0345 (C_5H_5O), 71.0504 (C_4H_7O), 69.0347 (C_4H_5O)
$C_9H_{14}O_5$-2		7.58	0.10	157.0869 ($C_8H_{13}O_3$), 139.0766 ($C_8H_{11}O_2$), 97.06660 (C_6H_9O), 85.0657 (C_5H_9O), 71.0138 ($C_3H_3O_2$), 57.0347 (C_3H_5O)
$C_9H_{14}O_5$-3		4.43	0.09	183.0659 ($C_9H_{11}O_4$), 157.0869 ($C_8H_{13}O_3$), 141.0557 ($C_7H_9O_3$), 139.0761 ($C_8H_{11}O_2$), 111.0813 ($C_7H_{11}O$), 57.0347 (C_3H_5O)
$C_9H_{14}O_6$-1	217.0716	7.41	0.02	169.0877 ($C_9H_{13}O_3$), 157.0504 ($C_7H_9O_4$), 153.0920 ($C_9H_{13}O_2$), 125.0974 ($C_8H_{13}O$), 113.0608 ($C_6H_9O_2$), 95.0502 (C_6H_7O), 85.0293 ($C_4H_5O_2$), 73.0296 ($C_3H_5O_2$), 59.0138 ($C_2H_3O_2$), 57.0344 (C_3H_5O)
$C_9H_{14}O_6$-2		7.03	0.06	199.0612 ($C_9H_{11}O_5$), 171.0659 ($C_8H_{11}O_4$), 155.0717 ($C_8H_{11}O_3$), 129.0923 ($C_7H_{13}O_2$), 127.0764 ($C_7H_{11}O_2$), 111.0817 ($C_7H_{11}O$), 99.0090 ($C_4H_3O_3$), 85.0661 (C_5H_9O), 59.0138 ($C_2H_3O_2$)

$C_9H_{14}O_6$-3		7.94	0.02	
$C_9H_{14}O_6$-4		10.49	0.03	
$C_9H_{14}O_6$-5		7.26	0.05	
$C_9H_{16}O_5$-1	203.0924	7.37	0.09	171.0661 ($C_8H_{11}O_4$), 127.0768 ($C_7H_{11}O_2$)
$C_9H_{16}O_5$-2		3.53	0.10	
$C_9H_{16}O_5$-3		8.4	0.05	
$C_9H_{16}O_6$	219.0873	8.35	0.40	99.0451 ($C_5H_7O_2$), 85.0295 ($C_4H_5O_2$), 67.0189 (C_4H_3O), 59.0138 ($C_2H_3O_2$), 57.0348 (C_3H_5O)
$C_{10}H_{14}O_4$-1	197.0819	6.83	0.17	97.0658 (C_6H_9O), 83.0502 (C_5H_7O), 71.0502 (C_4H_7O), 69.0346 (C_4H_5O), 57.0348 (C_3H_5O)
$C_{10}H_{14}O_4$-2		6.47	0.06	135.0817 ($C_9H_{11}O$), 125.0972 ($C_8H_{13}O$), 69.0346 (C_4H_5O), 57.0348 (C_3H_5O)
$C_{10}H_{14}O_4$-3		7.18	0.06	153.0917 ($C_9H_{13}O_2$), 135.0817 ($C_9H_{11}O$), 83.0502 (C_5H_7O), 69.0346 (C_4H_5O), 59.0138 ($C_2H_3O_2$)
$C_{10}H_{14}O_4$-4		8.58	0.04	179.0713 ($C_{10}H_{11}O_3$), 153.0922 ($C_9H_{13}O_2$), 57.0348 (C_3H_5O)
$C_{10}H_{14}O_5$-1	213.0768	6.64	1.57	195.0667 ($C_{10}H_{11}O_4$), 169.0877 ($C_9H_{13}O_3$), 151.0763 ($C_9H_{11}O_2$), 141.0922 ($C_8H_{13}O_2$), 139.07633 ($C_8H_{11}O_2$), 123.0812 ($C_8H_{11}O$), 111.0452 ($C_6H_7O_2$), 71.0504 (C_4H_7O), 57.0348 (C_3H_5O)
$C_{10}H_{14}O_5$-2		7.3	0.16	
$C_{10}H_{14}O_5$3		6.29	0.08	
$C_{10}H_{14}O_5$-4		9.05	0.04	

$C_{10}H_{14}O_6$-1	229.0717	7.44	0.18	185.0819 ($C_9H_{13}O_4$), 171.0659 ($C_8H_{11}O_4$), 167.0717 ($C_9H_{11}O_3$), 141.0922 ($C_8H_{13}O_2$), 123.0812 ($C_8H_{11}O$), 113.0611 ($C_6H_9O_2$), 85.0293 ($C_4H_5O_2$), 75.0090 ($C_2H_3O_3$), 71.0135 ($C_3H_3O_2$), 57.0348 (C_3H_5O)
$C_{10}H_{14}O_6$-2		6.36	0.04	
$C_{10}H_{16}O_3$-1	183.1027	10.98	0.55	183.1027 ($C_{10}H_{15}O_3$), 165.0921 ($C_{10}H_{13}O_2$), 141.0922 ($C_8H_{13}O_2$), 139.1127 ($C_9H_{15}O$), 123.0812 ($C_8H_{11}O$), 113.0611 ($C_6H_9O_2$), 97.0658 (C_6H_9O), 85.0658 (C_5H_9O), 69.0347 (C_4H_5O), 59.0138 ($C_2H_3O_2$), 57.0348 (C_3H_5O)
$C_{10}H_{16}O_3$-2		10.08	0.05	
$C_{10}H_{16}O_4$-1	199.0975	9.65	0.63	181.0869 ($C_{10}H_{13}O_3$), 163.0764 ($C_{10}H_{11}O_2$), 155.1079 ($C_9H_{15}O_2$), 153.0920 ($C_9H_{13}O_2$), 137.0973 ($C_9H_{13}O$), 125.0971 ($C_8H_{13}O$), 123.0812 ($C_8H_{11}O$), 109.0656 (C_7H_9O), 101.0606 ($C_5H_9O_2$), 95.0502 (C_6H_7O), 85.0294 ($C_4H_5O_2$), 81.0347 (C_5H_5O), 75.0087 ($C_2H_3O_3$), 73.0295 ($C_3H_5O_2$), 69.0345 (C_4H_5O), 59.0138 ($C_2H_3O_2$), 57.0348 (C_3H_5O)
$C_{10}H_{16}O_4$-2		7.73	0.65	181.0869 ($C_{10}H_{13}O_3$), 169.0877 ($C_9H_{13}O_3$), 153.0920 ($C_9H_{13}O_2$), 137.0971 ($C_9H_{13}O$), 135.0820 ($C_9H_{11}O$), 129.0558 ($C_6H_9O_3$), 121.0656 (C_8H_9O), 101.0242 ($C_4H_5O_3$), 97.0659 (C_6H_9O), 83.0502 (C_5H_7O), 69.0346 (C_4H_5O), 59.0138 ($C_2H_3O_2$), 57.0348 (C_3H_5O)
$C_{10}H_{16}O_4$-3		7.96	0.52	181.0869 ($C_{10}H_{13}O_3$), 141.0557 ($C_7H_9O_3$), 135.0816 ($C_9H_{11}O$), 129.0559 ($C_6H_9O_3$), 113.0608 ($C_6H_9O_2$), 101.0242 ($C_4H_5O_3$), 97.0658 (C_6H_9O), 85.0659 (C_5H_9O), 83.0501 (C_5H_7O), 71.0140 ($C_3H_3O_2$), 69.0346 (C_4H_5O), 59.0138 ($C_2H_3O_2$), 57.0348 (C_3H_5O)

$C_{10}H_{16}O_4$-4		6.43	0.18	
$C_{10}H_{16}O_4$-5		6.08	0.07	
$C_{10}H_{16}O_4$-6		8.4	0.08	
$C_{10}H_{16}O_4$-7		10.08	0.08	
$C_{10}H_{16}O_5$-1	215.0925	10.14	1.73	173.0828 ($C_8H_{13}O_4$), 157.0505 ($C_7H_9O_4$), 153.0920 ($C_9H_{13}O_2$), 141.0922 ($C_8H_{13}O_2$), 135.0820 ($C_9H_{11}O$), 123.0812 ($C_8H_{11}O$), 113.0607 ($C_6H_9O_2$), 111.0818 ($C_7H_{11}O$), 97.0294 ($C_5H_5O_2$), 95.0502 (C_6H_7O), 87.0452 ($C_4H_7O_2$), 85.0294 ($C_4H_5O_2$), 83.0502 (C_5H_7O), 73.0295 ($C_3H_5O_2$), 71.0139 ($C_3H_3O_2$), 69.0347 (C_4H_5O), 59.0138 ($C_2H_3O_2$), 57.0348 (C_3H_5O)
$C_{10}H_{16}O_5$-2		8.22	0.70	183.0659 ($C_9H_{11}O_4$), 171.1026 ($C_9H_{15}O_3$), 153.0920 ($C_9H_{13}O_2$), 127.1127 ($C_8H_{15}O$), 87.0451 ($C_4H_7O_2$), 85.0658 (C_5H_9O), 73.0295 ($C_3H_5O_2$), 59.0138 ($C_2H_3O_2$), 57.0348 (C_3H_5O)
$C_{10}H_{16}O_5$-3		7.48	0.33	169.0877 ($C_9H_{13}O_3$), 157.0504 ($C_7H_9O_4$), 153.0920 ($C_9H_{13}O_2$), 125.0974 ($C_8H_{13}O$), 113.0608 ($C_6H_9O_2$), 95.0502 (C_6H_7O), 85.0293 ($C_4H_5O_2$), 73.0296 ($C_3H_5O_2$), 59.0141 ($C_2H_3O_2$)
$C_{10}H_{16}O_5$-4		9.05	1.05	171.1026 ($C_9H_{15}O_3$), 169.0877 ($C_9H_{13}O_3$), 157.0872 ($C_8H_{13}O_3$), 153.0920 ($C_9H_{13}O_2$), 125.0970 ($C_8H_{13}O$), 99.0450 ($C_5H_7O_2$), 87.0087 ($C_3H_3O_3$), 85.0297 ($C_4H_5O_2$), 59.0138 ($C_2H_3O_2$), 57.0348 (C_3H_5O)
$C_{10}H_{16}O_5$-5		9.36	0.22	
$C_{10}H_{16}O_6$-1	231.0874	6.64	0.11	213.0768 ($C_{10}H_{13}O_5$), 195.0667 ($C_{10}H_{11}O_4$), 169.0872 ($C_9H_{13}O_3$), 151.0763 ($C_9H_{11}O_2$), 141.0924 ($C_8H_{13}O_2$), 139.0763 ($C_8H_{11}O_2$), 123.0812 ($C_8H_{11}O$), 111.0455 ($C_6H_7O_2$), 71.0502 (C_4H_7O), 57.0348 (C_3H_5O)

$C_{10}H_{16}O_6$-2		9.28	0.75	171.0671 ($C_8H_{11}O_4$), 127.0764 ($C_7H_{11}O_2$), 59.0138 ($C_2H_3O_2$)
$C_{10}H_{16}O_6$-3		5.06	0.81	189.0767 ($C_8H_{13}O_5$), 157.0504 ($C_7H_9O_4$), 109.0658 (C_7H_9O), 97.0658 (C_6H_9O), 85.0295 ($C_4H_5O_2$), 69.0345 (C_4H_5O), 59.0138 ($C_2H_3O_2$)
$C_{11}H_{16}O_6$-1	243.0874	11.03	0.40	185.0819 ($C_9H_{13}O_4$), 167.0717 ($C_9H_{11}O_3$), 141.0922 ($C_8H_{13}O_2$), 75.0088 ($C_2H_3O_3$), 71.0141 ($C_3H_3O_2$)
$C_{11}H_{16}O_6$-2		12.75	0.01	
$C_{12}H_{20}O_5$-1	243.1237	12.15	0.05	
$C_{12}H_{20}O_5$-2		12	0.04	
$C_{13}H_{20}O_6$-1	271.1184	13.37	0.09	185.0819 ($C_9H_{13}O_4$), 167.0717 ($C_9H_{11}O_3$), 141.0922 ($C_8H_{13}O_2$), 123.0812 ($C_8H_{11}O$), 99.0451($C_5H_7O_2$), 71.0138 ($C_3H_3O_2$), 57.0348 (C_3H_5O)
$C_{13}H_{20}O_6$-2		14.74	0.07	185.0819 ($C_9H_{13}O_4$), 167.0717 ($C_9H_{11}O_3$), 141.0922 ($C_8H_{13}O_2$), 123.0812 ($C_8H_{11}O$), 103.0400 ($C_4H_7O_3$), 85.0295 ($C_4H_5O_2$), 71.0139 ($C_3H_3O_2$), 57.0344 (C_3H_5O)
$C_{13}H_{20}O_6$-3		13.24	0.08	
$C_{13}H_{20}O_7$-1	287.1136	9.08	0.10	185.0819 ($C_9H_{13}O_4$), 167.0717 ($C_9H_{11}O_3$), 141.0922 ($C_8H_{13}O_2$), 123.0812 ($C_8H_{11}O$), 85.0295 ($C_4H_5O_2$), 71.0138 ($C_3H_3O_2$)
$C_{13}H_{20}O_7$-2		9.81	0.05	185.0819 ($C_9H_{13}O_4$), 167.0717 ($C_9H_{11}O_3$), 141.0922 ($C_8H_{13}O_2$), 85.0295 ($C_4H_5O_2$)
$C_{14}H_{22}O_6$-1	285.1331	19.95	0.01	211.0612 ($C_{10}H_{11}O_5$), 167.0717 ($C_9H_{11}O_3$)
$C_{14}H_{22}O_6$-2		11.28	0.03	
$C_{14}H_{20}O_8$	315.1084	10.24	0.44	185.0819 ($C_9H_{13}O_4$), 141.0922 ($C_8H_{13}O_2$), 129.0194 ($C_5H_5O_4$), 123.0819 ($C_8H_{11}O$), 111.0813 ($C_7H_{11}O$), 85.0295 ($C_4H_5O_2$), 71.0139 ($C_3H_3O_2$)

$C_{15}H_{22}O_6$-1	297.1344	12.56	0.02	
$C_{15}H_{22}O_6$-2		12.24	0.05	
$C_{15}H_{22}O_6$-3		12.09	0.04	
$C_{15}H_{22}O_6$-4		13.67	0.04	
$C_{15}H_{22}O_6$-5		12.8	0.02	
$C_{15}H_{24}O_5$-1	283.1549	15.5	0.07	143.0714 ($C_7H_{11}O_3$), 125.0610 ($C_7H_9O_2$), 85.0657 (C_5H_9O)
$C_{15}H_{24}O_5$-2		15.26	0.10	
$C_{15}H_{24}O_6$-1	299.1499	15.46	0.12	189.0768 ($C_8H_{13}O_5$), 171.0659 ($C_8H_{11}O_4$), 85.0657 (C_5H_9O), 71.0502 (C_4H_7O)
$C_{15}H_{24}O_6$-2		16.06	0.07	
$C_{15}H_{24}O_6$-3		17.24	0.03	
$C_{15}H_{24}O_6$-4		12.84	0.09	
$C_{15}H_{24}O_7$-1	315.1448	13.11	0.07	185.0819 ($C_9H_{13}O_4$), 167.0717 ($C_9H_{11}O_3$), 141.0922 ($C_8H_{13}O_2$), 129.0559 ($C_6H_9O_3$), 123.0812 ($C_8H_{11}O$), 85.0658 (C_5H_9O), 71.0139 ($C_3H_3O_2$), 57.0348 (C_3H_5O)
$C_{15}H_{24}O_7$-2		11.97	0.12	185.0819 ($C_9H_{13}O_4$), 171.0662 ($C_8H_{11}O_4$), 157.0872 ($C_8H_{13}O_3$), 143.0717 ($C_7H_{11}O_3$), 141.0924 ($C_8H_{13}O_2$), 127.0765 ($C_7H_{11}O_2$), 125.0761 ($C_7H_9O_2$), 109.0656 (C_7H_9O), 85.0658 (C_5H_9O)
$C_{15}H_{24}O_7$-3		11.71	0.03	185.0819 ($C_9H_{13}O_4$), 171.0659 ($C_8H_{11}O_4$), 167.0717 ($C_9H_{11}O_3$), 141.0922 ($C_8H_{13}O_2$), 127.0764 ($C_7H_{11}O_2$), 59.0138 ($C_2H_3O_2$)
$C_{15}H_{24}O_7$-4		13.01	0.03	

$C_{15}H_{24}O_8$-1	332.0309	13.53	0.09	261.0626 ($C_{10}H_{13}O_8$), 199.0611 ($C_9H_{11}O_5$), 189.0762 ($C_8H_{13}O_5$), 173.0451 ($C_7H_9O_5$), 171.0671 ($C_8H_{11}O_4$), 155.0715 ($C_8H_{11}O_3$), 145.0875 ($C_7H_{13}O_3$),137.0612 ($C_8H_9O_2$), 131.0350 ($C_5H_7O_4$), 129.0194 ($C_5H_5O_4$), 127.0760 ($C_7H_{11}O_2$),111.0813 ($C_7H_{11}O$), 95.0506 (C_6H_7O), 87.0449 ($C_4H_7O_2$), 85.0295 ($C_4H_5O_2$), 59.0138 ($C_2H_3O_2$), 57.0348 (C_3H_5O)
$C_{15}H_{24}O_8$-2		10.99	0.11	183.1027 ($C_{10}H_{15}O_3$), 171.0659 ($C_8H_{11}O_4$), 159.0669 ($C_7H_{11}O_4$), 127.0764 ($C_7H_{11}O_2$), 101.0246 ($C_4H_5O_3$), 97.0661 (C_6H_9O), 59.0138 ($C_2H_3O_2$), 57.0348 (C_3H_5O)
$C_{15}H_{24}O_8$-3		12.97	0.10	
$C_{15}H_{26}O_6$-1	301.1656	14.2	0.11	185.0819 ($C_9H_{13}O_4$), 141.0924 ($C_8H_{13}O_2$)
$C_{15}H_{26}O_6$-2		12.43	0.16	185.0819 ($C_9H_{13}O_4$), 167.0717 ($C_9H_{11}O_3$), 141.0922 ($C_8H_{13}O_2$), 129.0559 ($C_6H_9O_3$), 123.0812 ($C_8H_{11}O$), 97.0661 (C_6H_9O), 81.0345 (C_5H_5O), 57.0348 (C_3H_5O)
$C_{15}H_{26}O_6$-3		12.83	0.16	
$C_{15}H_{26}O_6$-4		13.75	0.06	
$C_{15}H_{26}O_6$-5		15.71	0.04	
$C_{15}H_{26}O_6$-6		16.36	0.03	
$C_{15}H_{26}O_6$-7		18.31	0.03	
$C_{16}H_{22}O_7$-1	325.1292	14.61	0.09	199.0974 ($C_{10}H_{15}O_4$), 185.0819 ($C_9H_{13}O_4$), 167.0717 ($C_9H_{11}O_3$), 157.0506 ($C_7H_9O_4$), 143.0349 ($C_6H_7O_4$), 141.0924 ($C_8H_{13}O_2$), 139.0398 ($C_7H_7O_3$), 123.0820 ($C_8H_{11}O$), 111.0452 ($C_6H_7O_2$), 97.295 ($C_5H_5O_2$), 85.0294 ($C_4H_5O_2$), 71.0138 ($C_3H_3O_2$), 59.0138 ($C_2H_3O_2$)
$C_{16}H_{22}O_7$-2		12.81	0.05	

$C_{16}H_{24}O_5$-1	295.1547	19.86	0.01	
$C_{16}H_{24}O_5$-2		13.21	0.03	
$C_{16}H_{24}O_5$-3		14.48	0.02	
$C_{16}H_{24}O_5$-4		12.95	0.02	
$C_{16}H_{24}O_6$-1	311.1501	13.52	2.39	185.0819 ($C_9H_{13}O_4$), 167.0717 ($C_9H_{11}O_3$), 143.0712 ($C_7H_{11}O_3$), 141.0922 ($C_8H_{13}O_2$), 125.0612 ($C_7H_9O_2$), 123.0812 ($C_8H_{11}O$), 85.0297 ($C_4H_5O_2$), 81.0345 (C_5H_5O), 71.0135 ($C_3H_3O_2$), 57.0348 (C_3H_5O)
$C_{16}H_{24}O_6$-2		14.76	0.13	
$C_{16}H_{24}O_6$-3		15.55	0.08	
$C_{16}H_{24}O_6$-4		17.02	0.05	
$C_{16}H_{24}O_6$-5		19.62	0.02	
$C_{16}H_{24}O_7$-1	327.1449	13.91	0.12	199.0974 ($C_{10}H_{15}O_4$), 185.0819 ($C_9H_{13}O_4$), 169.0872 ($C_9H_{13}O_3$), 167.0717 ($C_9H_{11}O_3$), 141.0924 ($C_8H_{13}O_2$), 123.0812 ($C_8H_{11}O$), 111.0813 ($C_7H_{11}O$), 85.0297 ($C_4H_5O_2$), 73.0296 ($C_3H_5O_2$), 57.0348 (C_3H_5O)
$C_{16}H_{24}O_7$-2		13.16	0.04	185.0819 ($C_9H_{13}O_4$), 171.0659 ($C_8H_{11}O_4$), 155.0717 ($C_8H_{11}O_3$), 111.0816 ($C_7H_{11}O$), 85.0659 (C_5H_9O)
$C_{16}H_{24}O_7$-3		11.11	0.14	
$C_{16}H_{24}O_7$-4		11.89	0.05	
$C_{16}H_{24}O_8$-1	343.1399	13.78	0.70	185.0819 ($C_9H_{13}O_4$), 167.0717 ($C_9H_{11}O_3$), 157.0505 ($C_7H_9O_4$), 141.0922 ($C_8H_{13}O_2$), 123.0812 ($C_8H_{11}O$), 113.0611 ($C_6H_9O_2$), 71.0139 ($C_3H_3O_2$)
$C_{16}H_{24}O_8$ -2		11.82	0.07	189.0768 ($C_8H_{13}O_5$), 171.0659 ($C_8H_{11}O_4$), 127.0765 ($C_7H_{11}O_2$)

$C_{16}H_{24}O_8$ -3		13.39	0.03	
$C_{16}H_{26}O_5$-1	297.1711	16.85	0.05	
$C_{16}H_{26}O_5$-2		17.09	0.02	
$C_{16}H_{26}O_5$-3		13.01	0.01	
$C_{16}H_{26}O_6$-1	313.1655	16.78	2.20	185.0819 ($C_9H_{13}O_4$), 167.0717 ($C_9H_{11}O_3$), 141.0922 ($C_8H_{13}O_2$), 123.0812 ($C_8H_{11}O$), 99.0451($C_5H_7O_2$), 71.0138 ($C_3H_3O_2$), 57.0348 (C_3H_5O)
$C_{16}H_{26}O_6$-2		18.47	0.26	171.0661 ($C_8H_{11}O_4$), 141.0922 ($C_8H_{13}O_2$), 127.0764 ($C_7H_{11}O_2$), 109.0658 (C_7H_9O)
$C_{16}H_{26}O_6$-3		16.05	0.12	157.0869 ($C_8H_{13}O_3$), 155.0713 ($C_8H_{11}O_3$), 125.0609 ($C_7H_9O_2$), 111.0816 ($C_7H_{11}O$), 71.0502 (C_4H_7O)
$C_{16}H_{26}O_6$-4		15.05	0.10	171.0661 ($C_8H_{11}O_4$), 157.0504 ($C_7H_9O_4$), 139.1131 ($C_9H_{15}O$), 127.0764 ($C_7H_{11}O_2$), 113.0608 ($C_6H_9O_2$), 71.0503 (C_4H_7O)
$C_{16}H_{26}O_6$-5		18.01	0.16	
$C_{16}H_{26}O_7$-1	329.1604	15.22	0.77	285.1709 ($C_{15}H_{25}O_5$), 267.1602 ($C_{15}H_{23}O_4$), 171.0662 ($C_8H_{11}O_4$), 157.0869 ($C_8H_{13}O_3$), 145.0870 ($C_7H_{13}O_3$), 127.0765 ($C_7H_{11}O_2$), 109.0657 (C_7H_9O), 85.0658 (C_5H_9O), 71.0502 (C_4H_7O), 59.0138 ($C_2H_3O_2$)
$C_{16}H_{26}O_7$-2		14.88	0.19	171.0661 ($C_8H_{11}O_4$), 157.0874 ($C_8H_{13}O_3$), 145.0870 ($C_7H_{13}O_3$), 141.0922 ($C_8H_{13}O_2$), 127.0764 ($C_7H_{11}O_2$), 111.0817 ($C_7H_{11}O$), 85.0658 (C_5H_9O)
$C_{16}H_{26}O_7$-3		14.22	0.12	201.0769 ($C_9H_{13}O_5$), 185.0819 ($C_9H_{13}O_4$), 167.0717 ($C_9H_{11}O_3$), 141.0922 ($C_8H_{13}O_2$), 123.0812 ($C_8H_{11}O$), 57.0348 (C_3H_5O)
$C_{16}H_{26}O_7$-4		13.35	0.12	185.0819 ($C_9H_{13}O_4$), 167.0717 ($C_9H_{11}O_3$), 157.0872 ($C_8H_{13}O_3$), 141.0922 ($C_8H_{13}O_2$), 123.0812 ($C_8H_{11}O$),

				111.0817 ($C_7H_{11}O$), 99.0454 ($C_5H_7O_2$), 71.0142 ($C_3H_3O_2$), 57.0348 (C_3H_5O)
$C_{16}H_{26}O_7$-5		12.54	0.14	185.0819 ($C_9H_{13}O_4$), 171.0659 ($C_8H_{11}O_4$), 157.0872 ($C_8H_{13}O_3$), 127.0764 ($C_7H_{11}O_2$), 111.0813 ($C_7H_{11}O$)
$C_{16}H_{26}O_7$-6		13.17	0.10	185.0819 ($C_9H_{13}O_4$), 167.0717 ($C_9H_{11}O_3$), 141.0922 ($C_8H_{13}O_2$), 123.0812 ($C_8H_{11}O$), 99.0451($C_5H_7O_2$), 71.0138 ($C_3H_3O_2$)
$C_{16}H_{26}O_7$-7		15.7	0.04	
$C_{16}H_{26}O_7$-8		16.84	0.07	
$C_{16}H_{26}O_8$-1	345.1555	13.21	0.05	189.0768 ($C_8H_{13}O_5$), 185.0819 ($C_9H_{13}O_4$), 169.0872 ($C_9H_{13}O_3$), 141.0922 ($C_8H_{13}O_2$), 127.0764 ($C_7H_{11}O_2$), 85.0658 (C_5H_9O)
$C_{16}H_{26}O_8$-2		12.86	0.05	189.0768 ($C_8H_{13}O_5$), 171.0659 ($C_8H_{11}O_4$), 145.0870 ($C_7H_{13}O_3$), 127.0764 ($C_7H_{11}O_2$), 101.0974 ($C_6H_{13}O$), 85.0658 (C_5H_9O), 57.0348 (C_3H_5O)
$C_{16}H_{26}O_8$-3		13.8	0.05	
$C_{16}H_{26}O_8$-4		15.82	0.04	
$C_{16}H_{26}O_8$-5		11.14	0.04	
$C_{16}H_{26}O_8$-6		11.57	0.04	
$C_{16}H_{26}O_9$-1	361.1504	14.47	0.09	
$C_{16}H_{26}O_9$-2		12.39	0.07	
$C_{16}H_{26}O_9$-3		12.07	0.04	
$C_{16}H_{26}O_9$-4		12.65	0.03	
$C_{17}H_{24}O_6$-1	323.1498	16.72	0.05	

$C_{17}H_{24}O_6$-2		16.18	0.02	
$C_{17}H_{26}O_5$-1	309.1704	15.44	0.13	221.1915 ($C_{15}H_{25}O$), 167.0717 ($C_9H_{11}O_3$), 141.0922 ($C_8H_{13}O_2$), 139.1127 ($C_9H_{15}O$), 123.0812 ($C_8H_{11}O$), 113.0611 ($C_6H_9O_2$), 99.0454 ($C_5H_7O_2$), 57.0348 (C_3H_5O)
$C_{17}H_{26}O_5$-2		15.76	0.39	
$C_{17}H_{26}O_5$-3		18.99	0.05	
$C_{17}H_{26}O_5$-4		19.36	0.02	
$C_{17}H_{26}O_6$-1	325.1654	16.56	0.56	185.0819 ($C_9H_{13}O_4$), 167.0717 ($C_9H_{11}O_3$), 141.0922 ($C_8H_{13}O_2$), 123.0812 ($C_8H_{11}O$), 57.0348 (C_3H_5O)
$C_{17}H_{26}O_6$-2		14.46	0.10	
$C_{17}H_{26}O_6$-3		12.39	0.05	
$C_{17}H_{26}O_6$-4		11.87	0.04	
$C_{17}H_{26}O_7$-1	341.1602	14.09	0.77	211.1338 ($C_{12}H_{19}O_3$),185.0819 ($C_9H_{13}O_4$), 171.1025 ($C_9H_{15}O_3$), 169.0876 ($C_9H_{13}O_3$), 167.0717 ($C_9H_{11}O_3$), 141.0924 ($C_8H_{13}O_2$), 127.0765 ($C_7H_{11}O_2$), 111.0087 ($C_5H_3O_3$), 85.0658 (C_5H_9O), 85.0297 ($C_4H_5O_2$), 69.0346 (C_4H_5O), 59.0138 ($C_2H_3O_2$)
$C_{17}H_{26}O_7$-2		15.05	0.23	185.0819 ($C_9H_{13}O_4$), 167.0717 ($C_9H_{11}O_3$), 155.1079 ($C_9H_{15}O_2$), 141.0922 ($C_8H_{13}O_2$), 111.0813 ($C_7H_{11}O$), 71.0138 ($C_3H_3O_2$)
$C_{17}H_{26}O_7$-3		13.47	0.06	185.0819 ($C_9H_{13}O_4$), 171.0659 ($C_8H_{11}O_4$), 169.0872 ($C_9H_{13}O_3$), 127.0764 ($C_7H_{11}O_2$), 111.0813 ($C_7H_{11}O$), 73.0298 ($C_3H_5O_2$), 59.0138 ($C_2H_3O_2$)
$C_{17}H_{26}O_7$-4		13.17	0.17	221.1176 ($C_{13}H_{17}O_3$), 185.0819 ($C_9H_{13}O_4$), 177.1293 ($C_{12}H_{17}O$), 171.0659 ($C_8H_{11}O_4$), 141.0924 ($C_8H_{13}O_2$), 127.0765 ($C_7H_{11}O_2$), 84.0454 ($C_4H_7O_2$), 71.0142 ($C_3H_3O_2$), 59.0141 ($C_2H_3O_2$)

$C_{17}H_{26}O_7$-5		16.28	0.06	
$C_{17}H_{26}O_8$-1	357.1552	13.92	0.04	185.0819 ($C_9H_{13}O_4$), 171.0659 ($C_8H_{11}O_4$), 167.0717 ($C_9H_{11}O_3$), 141.0922 ($C_8H_{13}O_2$), 127.0764 ($C_7H_{11}O_2$), 109.0660 (C_7H_9O), 99.0454 ($C_5H_7O_2$), 71.0142 ($C_3H_3O_2$)
$C_{17}H_{26}O_8$-2		15.16	0.39	185.0819 ($C_9H_{13}O_4$), 171.0659 ($C_8H_{11}O_4$), 167.0717 ($C_9H_{11}O_3$), 141.0922 ($C_8H_{13}O_2$), 127.0764 ($C_7H_{11}O_2$), 109.0660 (C_7H_9O), 85.0658 (C_5H_9O), 59.0138 ($C_2H_3O_2$)
$C_{17}H_{26}O_8$-3		14.26	0.08	185.0819 ($C_9H_{13}O_4$), 171.0659 ($C_8H_{11}O_4$), 167.0717 ($C_9H_{11}O_3$), 141.0922 ($C_8H_{13}O_2$), 127.0764 ($C_7H_{11}O_2$), 97.0661 (C_6H_9O), 85.0297 ($C_4H_5O_2$), 69.0347 (C_4H_5O)
$C_{17}H_{28}O_5$-1	311.1862	17.17	0.06	
$C_{17}H_{28}O_5$-2		20.64	0.03	
$C_{17}H_{28}O_5$-3		15.92	0.03	
$C_{17}H_{28}O_7$-1	343.1759	16.37	0.23	189.0768 ($C_8H_{13}O_5$), 173.0459 ($C_7H_9O_5$), 145.0870 ($C_7H_{13}O_3$), 131.0348 ($C_5H_7O_4$), 127.0768 ($C_7H_{11}O_2$), 87.0450 ($C_4H_7O_2$), 85.0658 (C_5H_9O), 69.0347 (C_4H_5O), 57.0348 (C_3H_5O)
$C_{17}H_{28}O_7$-2		13.37	0.06	185.0819 ($C_9H_{13}O_4$), 171.0659 ($C_8H_{11}O_4$), 141.0922 ($C_8H_{13}O_2$), 127.0764 ($C_7H_{11}O_2$), 123.0812 ($C_8H_{11}O$), 113.0611 ($C_6H_9O_2$), 109.0659 (C_7H_9O), 85.0297 ($C_4H_5O_2$), 73.0296 ($C_3H_5O_2$)
$C_{17}H_{28}O_7$-3		14.52	0.07	185.0819 ($C_9H_{13}O_4$), 171.0659 ($C_8H_{11}O_4$), 167.0717 ($C_9H_{11}O_3$), 141.0922 ($C_8H_{13}O_2$), 127.0764 ($C_7H_{11}O_2$), 59.0138 ($C_2H_3O_2$)

$C_{18}H_{26}O_5$-1	321.1705	17.5	0.06	209.1552 ($C_{13}H_{21}O_2$), 177.1285 ($C_{12}H_{17}O$), 153.0920 ($C_9H_{13}O_2$), 123.0812 ($C_8H_{11}O$), 97.0660 (C_6H_9O), 69.0345 (C_4H_5O)
$C_{18}H_{26}O_5$-2		17.79	0.07	
$C_{18}H_{26}O_6$-1	337.1654	14.96	0.12	231.1765 ($C_{16}H_{23}O$), 213.0775 ($C_{10}H_{13}O_5$), 185.0819 ($C_9H_{13}O_4$), 167.0717 ($C_9H_{11}O_3$), 155.0717 ($C_8H_{11}O_3$), 141.0924 ($C_8H_{13}O_2$), 123.0812 ($C_8H_{11}O$), 59.0138 ($C_2H_3O_2$)
$C_{18}H_{26}O_6$-2		17.32	0.29	249.1849 ($C_{16}H_{25}O_2$), 231.1753 ($C_{16}H_{23}O$), 213.0768 ($C_{10}H_{13}O_6$), 211.0977 ($C_{11}H_{15}O_4$), 195.0662 ($C_{10}H_{11}O_4$), 169.0870 ($C_9H_{13}O_3$), 163.1127 ($C_{11}H_{15}O$), 151.0765 ($C_9H_{11}O_2$), 141.0921 ($C_8H_{13}O_2$), 125.0608 ($C_7H_9O_2$), 123.0815 ($C_8H_{11}O$), 121.06008 (C_8H_9O), 109.0660 (C_7H_9O), 95.0504 (C_6H_7O), 59.0136 ($C_2H_3O_2$), 57.0348 (C_3H_5O)
$C_{18}H_{26}O_6$-3		17.66	0.65	
$C_{18}H_{26}O_7$-1	353.1604	14.56	0.32	199.0974 ($C_{10}H_{15}O_4$), 185.0819 ($C_9H_{13}O_4$), 171.1025 ($C_9H_{15}O_3$), 167.0717 ($C_9H_{11}O_3$), 141.0924 ($C_8H_{13}O_2$), 127.0765 ($C_7H_{11}O_2$), 123.0812 ($C_8H_{11}O$), 85.0297 ($C_4H_5O_2$), 73.0296 ($C_3H_5O_2$)
$C_{18}H_{26}O_7$-2		13.34	0.12	199.0974 ($C_{10}H_{15}O_4$), 185.0819 ($C_9H_{13}O_4$), 171.1025 ($C_9H_{15}O_3$), 167.0717 ($C_9H_{11}O_3$), 141.0924 ($C_8H_{13}O_2$), 127.0765 ($C_7H_{11}O_2$), 123.0812 ($C_8H_{11}O$), 109.0656 (C_7H_9O), 85.0297 ($C_4H_5O_2$), 73.0296 ($C_3H_5O_2$)
$C_{18}H_{28}O_4$-1	307.1914	16.59	0.19	193.1604 ($C_{13}H_{21}O$), 165.1290 ($C_{11}H_{17}O$), 141.0922 ($C_8H_{13}O_2$), 123.0812 ($C_8H_{11}O$), 111.0813 ($C_7H_{11}O$), 111.0450 ($C_8H_7O_2$), 85.0658 (C_5H_9O), 83.0503 (C_5H_7O), 57.0348 (C_3H_5O)
$C_{18}H_{28}O_4$-2		17.04	0.12	237.1505 ($C_{14}H_{21}O_3$), 199.1702 ($C_{12}H_{23}O_2$), 111.0815 ($C_7H_{11}O$), 85.0661 (C_5H_9O), 57.0348 (C_3H_5O)

$C_{18}H_{28}O_5$-1	323.1863	17.76	0.41	185.0819 ($C_9H_{13}O_4$), 167.0717 ($C_9H_{11}O_3$), 155.1079 ($C_9H_{15}O_2$), 141.0924 ($C_8H_{13}O_2$), 123.0812 ($C_8H_{11}O$), 81.0346 (C_5H_5O), 69.0345 (C_4H_5O), 57.0348 (C_3H_5O)
$C_{18}H_{28}O_5$-2		15.6	0.06	167.1079 $C_{10}H_{15}O_2$), 139.1127 ($C_9H_{15}O$), 111.0815 ($C_7H_{11}O$), 85.0661 (C_5H_9O), 69.0346 (C4H5O), 59.0138 ($C_2H_3O_2$)
$C_{18}H_{28}O_5$-3		18.3	0.08	
$C_{18}H_{28}O_5$-4		19.99	0.05	
$C_{18}H_{28}O_6$-1	339.1813	17.97	0.17	185.0819 ($C_9H_{13}O_4$), 167.0717 ($C_9H_{11}O_3$), 153.0920 ($C_9H_{13}O_2$), 141.0924 ($C_8H_{13}O_2$), 123.0812 ($C_8H_{11}O$), 71.0139 ($C_3H_3O_2$), 57.0348 (C_3H_5O)
$C_{18}H_{28}O_6$-2		15.82	0.16	215.0926 ($C_{10}H_{15}O_5$), 199.0974 ($C_{10}H_{15}O_4$), 185.0819 ($C_9H_{13}O_4$), 181.0870 ($C_{10}H_{13}O_3$), 157.0870 ($C_8H_{13}O_3$), 141.0924 ($C_8H_{13}O_2$), 123.0812 ($C_8H_{11}O$), 85.0297 ($C_4H_5O_2$), 73.0296 ($C_3H_5O_2$)
$C_{18}H_{28}O_6$-3		15.29	0.17	
$C_{18}H_{28}O_6$-4		14.12	0.11	
$C_{18}H_{28}O_6$-5		16.99	0.04	
$C_{18}H_{28}O_6$-6		18.72	0.01	
$C_{18}H_{28}O_7$-1	355.1761	14.5	1.19	199.0974 ($C_{10}H_{15}O_4$), 185.0819 ($C_9H_{13}O_4$), 167.0717 ($C_9H_{11}O_3$), 141.0924 ($C_8H_{13}O_2$), 111.0813 ($C_7H_{11}O$), 99.0450 ($C_5H_7O_2$), 71.0139 ($C_3H_3O_2$), 57.0348 (C_3H_5O)
$C_{18}H_{28}O_7$-2		14.68	0.23	199.0974 ($C_{10}H_{15}O_4$), 185.0819 ($C_9H_{13}O_4$), 181.0870 ($C_{10}H_{13}O_3$), 167.0717 ($C_9H_{11}O_3$), 155.0717 (C8H11O3), 141.0924 ($C_8H_{13}O_2$), 123.0812 ($C_8H_{11}O$), 111.0813 ($C_7H_{11}O$), 109.0657 (C_7H_9O), 85.0297 ($C_4H_5O_2$), 73.0295 ($C_3H_5O_2$)

$C_{18}H_{28}O_7$-3		16.01	0.11	237.1132 ($C_{13}H_{17}O_4$), 215.0922 ($C_{10}H_{15}O_5$), 185.0819 ($C_9H_{13}O_4$), 167.0717 ($C_9H_{11}O_3$), 141.0924 ($C_8H_{13}O_2$), 137.0968 ($C_9H_{13}O$), 123.0812 ($C_8H_{11}O$), 111.0455 ($C_6H_7O_2$), 97.0656 (C_6H_9O), 81.0347 (C_5H_5O), 71.0140 ($C_3H_3O_2$), 59.0138 ($C_2H_3O_2$)
$C_{18}H_{28}O_7$-4		15.29	0.13	
$C_{18}H_{28}O_7$-5		14.84	0.09	
$C_{18}H_{30}O_4$-1	309.2071	21.48	0.03	169.1238 ($C_{10}H_{17}O_2$), 151.1130 ($C_{10}H_{15}O$)
$C_{18}H_{30}O_4$-2		21.07	0.03	
$C_{18}H_{30}O_4$-3		16.81	0.01	
$C_{18}H_{30}O_4$-4		17.41	0.02	
$C_{18}H_{30}O_5$-1	325.2014	15.9	0.06	
$C_{18}H_{30}O_5$-2		17.4	0.03	
$C_{18}H_{30}O_5$-3		16.67	0.04	
$C_{18}H_{30}O_7$-1	357.1916	19.99	0.11	189.0768 ($C_8H_{13}O_5$), 171.0659 ($C_8H_{11}O_4$), 169.1238 ($C_{10}H_{17}O_2$), 145.0870 ($C_7H_{13}O_3$), 85.0659 (C_5H_9O)
$C_{18}H_{30}O_7$-2		19.32	0.08	189.0768 ($C_8H_{13}O_5$), 171.0659 ($C_8H_{11}O_4$), 169.1238 ($C_{10}H_{17}O_2$), 151.1129 ($C_{10}H_{15}O$), 145.0870 ($C_7H_{13}O_3$), 131.0349 ($C_5H_7O_4$), 127.0765 ($C_7H_{11}O_2$), 101.0972 ($C_6H_{13}O$), 87.0453 ($C_4H_7O_2$), 85.0659 (C_5H_9O), 69.0346 (C_4H_5O), 57.0348 (C_3H_5O)
$C_{18}H_{30}O_8$-1	373.1865	14.06	0.93	
$C_{18}H_{30}O_8$-2		15.05	0.04	
$C_{18}H_{30}O_8$-3		16.31	0.03	
$C_{18}H_{30}O_8$-4		18.07	0.01	

$C_{18}H_{30}O_9$-1	389.1816	16.69	0.15	189.0768 ($C_8H_{13}O_5$), 171.0659 ($C_8H_{11}O_4$), 145.0870 ($C_7H_{13}O_3$), 127.0764 ($C_7H_{11}O_2$), 125.0612 ($C_7H_9O_2$), 107.0502 (C_7H_7O), 85.0658 (C_5H_9O), 57.0348 (C_3H_5O)
$C_{18}H_{30}O_9$-2		17.65	0.01	
$C_{19}H_{28}O_6$-1	351.1811	16.1	0.70	199.0974 ($C_{10}H_{15}O_4$), 185.0819 ($C_9H_{13}O_4$), 169.0872 ($C_9H_{13}O_3$), 167.0717 ($C_9H_{11}O_3$), 141.0924 ($C_8H_{13}O_2$), 125.0972 ($C_8H_{13}O$), 123.0812 ($C_8H_{11}O$), 101.0608 ($C_5H_9O_2$), 85.0294 ($C_4H_5O_2$), 69.0347 (C4H5O), 57.0348 (C_3H_5O)
$C_{19}H_{28}O_6$-2		17.28	0.09	195.0668 ($C_{10}H_{11}O_4$), 185.0819 ($C_9H_{13}O_4$), 167.0717 ($C_9H_{11}O_3$), 141.0924 ($C_8H_{13}O_2$), 123.0812 ($C_8H_{11}O$), 107.0504 (C_7H_7O), 85.0298 ($C_4H_5O_2$), 57.0348 (C_3H_5O)
$C_{19}H_{28}O_6$-3		17.72	0.10	185.0819 ($C_9H_{13}O_4$), 167.0717 ($C_9H_{11}O_3$), 141.0924 ($C_8H_{13}O_2$),123.0812 ($C_8H_{11}O$)
$C_{19}H_{28}O_6$-4		15.56	0.11	185.0819 ($C_9H_{13}O_4$), 183.1027 ($C_{10}H_{15}O_3$), 169.0872 ($C_9H_{13}O_3$), 167.0717 ($C_9H_{11}O_3$), 141.0924 ($C_8H_{13}O_2$), 125.0972 ($C_8H_{13}O$), 123.0812 ($C_8H_{11}O$), 113.0610 ($C_6H_9O_2$), 59.0141 ($C_2H_3O_2$), 57.0348 (C_3H_5O)
$C_{19}H_{28}O_7$-1	367.1760	15.88	4.80	199.0974 ($C_{10}H_{15}O_4$), 185.0819 ($C_9H_{13}O_4$), 167.0717 ($C_9H_{11}O_3$), 141.0924 ($C_8H_{13}O_2$), 123.0812 ($C_8H_{11}O$), 99.0450 ($C_5H_7O_2$), 71.0139 ($C_3H_3O_2$)
$C_{19}H_{28}O_7$-2		15.18	0.24	199.0974 ($C_{10}H_{15}O_4$), 185.0819 ($C_9H_{13}O_4$), 167.0717 ($C_9H_{11}O_3$), 141.0924 ($C_8H_{13}O_2$), 123.0812 ($C_8H_{11}O$), 71.0139 ($C_3H_3O_2$), 57.0348 (C_3H_5O)
$C_{19}H_{28}O_7$-3		17.02	0.11	199.0974 ($C_{10}H_{15}O_4$), 185.0819 ($C_9H_{13}O_4$), 171.1025 ($C_9H_{15}O_3$), 167.0717 ($C_9H_{11}O_3$), 157.0870 ($C_8H_{13}O_3$), 141.0924 ($C_8H_{13}O_2$), 123.0812 ($C_8H_{11}O$), 85.0297 ($C_4H_5O_2$), 59.0141 ($C_2H_3O_2$)

$C_{19}H_{28}O_7$-4		17.15	0.10	199.0974 ($C_{10}H_{15}O_4$), 185.0819 ($C_9H_{13}O_4$), 181.0870 ($C_{10}H_{13}O_3$), 169.0876 ($C_9H_{13}O_3$), 167.0717 ($C_9H_{11}O_3$), 157.0867 ($C_8H_{13}O_3$), 139.0763 ($C_8H_{11}O_2$), 137.0970 ($C_9H_{13}O$), 123.0820 ($C_8H_{11}O$), 73.0296 ($C_3H_5O_2$), 59.0141 ($C_2H_3O_2$)
$C_{19}H_{28}O_7$-5		15.36	0.15	199.0974 ($C_{10}H_{15}O_4$), 185.0819 ($C_9H_{13}O_4$), 167.0717 ($C_9H_{11}O_3$), 153.0916 ($C_9H_{13}O_2$), 141.0924 ($C_8H_{13}O_2$), 137.0968 ($C_9H_{13}O$), 99.0454 ($C_5H_7O_2$), 57.0348 (C_3H_5O)
$C_{19}H_{30}O_5$-1	337.2019	15.36	1.53	213.1133 ($C_{11}H_{17}O_4$), 195.1027 ($C_{11}H_{15}O_3$), 183.1026 ($C_{10}H_{15}O_3$), 169.0868 ($C_9H_{13}O_3$), 167.1080 ($C_{10}H_{15}O_2$), 153.0919 ($C_9H_{13}O_2$), 141.0920 ($C_8H_{13}O_2$), 125.0972 ($C_8H_{13}O$), 123.0812 ($C_8H_{11}O$), 97.0295 ($C_5H_5O_2$), 85.0658 (C_5H_9O), 71.0139 ($C_3H_3O_2$), 69.0346 (C_4H_5O), 59.0136 ($C_2H_3O_2$), 57.0348 (C_3H_5O)
$C_{19}H_{30}O_5$-2		20.24	0.12	185.0819 ($C_9H_{13}O_4$), 169.1234 ($C_{10}H_{17}O_2$), 167.0717 ($C_9H_{11}O_3$), 151.1123 ($C_{10}H_{15}O$), 141.0924 ($C_8H_{13}O_2$), 123.0812 ($C_8H_{11}O$), 81.0347 (C_5H_5O)
$C_{19}H_{30}O_5$-3		21.05	0.04	185.0819 ($C_9H_{13}O_4$), 167.0717 ($C_9H_{11}O_3$), 153.0928 ($C_9H_{13}O_2$), 141.0924 ($C_8H_{13}O_2$), 123.0812 ($C_8H_{11}O$)
$C_{19}H_{30}O_5$-4		15.57	0.50	
$C_{19}H_{30}O_5$-5		20.08	0.06	
$C_{19}H_{30}O_5$-6		16.67	0.08	
$C_{19}H_{30}O_6$-1	353.1968	16.21	0.37	185.0819 ($C_9H_{13}O_4$), 167.0717 ($C_9H_{11}O_3$), 141.0924 ($C_8H_{13}O_2$), 123.0812 ($C_8H_{11}O$), 109.0658 (C_7H_9O), 81.0346 (C_5H_5O), 57.0348 (C_3H_5O)
$C_{19}H_{30}O_6$-2		16.5	0.07	185.0819 ($C_9H_{13}O_4$), 167.0717 ($C_9H_{11}O_3$), 141.0924 ($C_8H_{13}O_2$), 123.0812 ($C_8H_{11}O$), 59.0138 ($C_2H_3O_2$)

$C_{19}H_{30}O_6$-3		17.86	0.17	185.1181 ($C_{10}H_{17}O_3$), 167.0717 ($C_9H_{11}O_3$), 141.0924 ($C_8H_{13}O_2$), 123.0812 ($C_8H_{11}O$)
$C_{19}H_{30}O_6$-4		16.64	0.27	223.1136 ($C_{13}H_{19}O_3$), 213.0761 ($C_{10}H_{13}O_5$), 185.0819 ($C_9H_{13}O_4$), 167.0717 ($C_9H_{11}O_3$), 141.0924 ($C_8H_{13}O_2$), 139.1130 ($C_9H_{15}O$), 123.0812 ($C_8H_{11}O$), 59.0138 ($C_2H_3O_2$), 57.0348 (C_3H_5O)
$C_{19}H_{30}O_6$-5		15.27	0.08	185.0819 ($C_9H_{13}O_4$), 167.0717 ($C_9H_{11}O_3$), 141.0924 ($C_8H_{13}O_2$), 123.0812 ($C_8H_{11}O$), 109.0658 (C_7H_9O), 71.0141 ($C_3H_3O_2$)
$C_{19}H_{30}O_6$-6		20.11	0.06	
$C_{19}H_{30}O_6$-7		15.93	0.05	
$C_{19}H_{30}O_7$-1	369.1918	16.95	0.50	185.0819 ($C_9H_{13}O_4$), 167.0717 ($C_9H_{11}O_3$), 141.0924 ($C_8H_{13}O_2$), 123.0812 ($C_8H_{11}O$), 99.0454 ($C_5H_7O_2$), 71.0138 ($C_3H_3O_2$)
$C_{19}H_{30}O_7$-2		14.74	0.25	211.0965 ($C_{11}H_{15}O_4$), 185.0819 ($C_9H_{13}O_4$), 141.0924 ($C_8H_{13}O_2$)
$C_{19}H_{30}O_7$-3		18.64	0.06	185.0819 ($C_9H_{13}O_4$), 167.0717 ($C_9H_{11}O_3$), 141.0924 ($C_8H_{13}O_2$), 123.0812 ($C_8H_{11}O$), 87.0451 ($C_4H_7O_2$), 71.0138 ($C_3H_3O_2$), 59.0138 ($C_2H_3O_2$)
$C_{19}H_{30}O_7$-4		17.26	0.06	185.0819 ($C_9H_{13}O_4$), 167.0717 ($C_9H_{11}O_3$), 141.0924 ($C_8H_{13}O_2$), 123.0812 ($C_8H_{11}O$)
$C_{19}H_{30}O_8$-1	385.1865	13.08	0.16	241.1080 ($C_{12}H_{17}O_5$), 213.1143 ($C_{11}H_{17}O_4$), 199.0974 ($C_{10}H_{15}O_4$), 185.0819 ($C_9H_{13}O_4$), 167.0717 ($C_9H_{11}O_3$), 141.0924 ($C_8H_{13}O_2$), 123.0812 ($C_8H_{11}O$), 85.0297 ($C_4H_5O_2$), 73.0296 ($C_3H_5O_2$)
$C_{19}H_{30}O_8$-2		13.88	0.14	213.0767 ($C_{10}H_{13}O_5$), 185.0819 ($C_9H_{13}O_4$), 141.0924 ($C_8H_{13}O_2$)
$C_{19}H_{30}O_8$-3		14.91	0.13	267.1246 ($C_{14}H_{19}O_5$), 197.0819 ($C_{10}H_{13}O_4$), 185.0819 ($C_9H_{13}O_4$), 153.0916 ($C_9H_{13}O_2$), 141.0924 ($C_8H_{13}O_2$),

				123.0812 ($C_8H_{11}O$), 95.0503 (C_6H_7O), 81.0349 (C_5H_5O), 69.0345 (C_4H_5O), 59.0138 ($C_2H_3O_2$), 57.0348 (C_3H_5O)
$C_{19}H_{30}O_8$-4		14.82	0.09	267.1246 ($C_{14}H_{19}O_5$), 197.0819 ($C_{10}H_{13}O_4$), 185.0819 ($C_9H_{13}O_4$), 153.0916 ($C_9H_{13}O_2$), 123.0812 ($C_8H_{11}O$), 69.0345 (C_4H_5O), 59.0138 ($C_2H_3O_2$)
$C_{19}H_{30}O_8$-5		15.96	0.09	215.0926 ($C_{10}H_{15}O_5$), 185.0819 ($C_9H_{13}O_4$), 169.0872 ($C_9H_{13}O_3$), 167.0717 ($C_9H_{11}O_3$), 141.0924 ($C_8H_{13}O_2$), 123.0812 ($C_8H_{11}O$), 99.0454 ($C_5H_7O_2$), 59.0138 ($C_2H_3O_2$)
$C_{19}H_{30}O_8$-6		15.19	0.17	
$C_{19}H_{28}O_9$-1	399.1660	14.06	0.13	185.0819 ($C_9H_{13}O_4$), 141.0924 ($C_8H_{13}O_2$)
$C_{19}H_{28}O_9$-2		14.19	0.10	185.0819 ($C_9H_{13}O_4$), 167.0717 ($C_9H_{11}O_3$), 141.0924 ($C_8H_{13}O_2$), 115.0402 ($C_5H_7O_3$), 71.0142 ($C_3H_3O_2$), 57.0348 (C_3H_5O)
$C_{19}H_{28}O_9$-3		14.82	0.37	199.0974 ($C_{10}H_{15}O_4$), 185.0819 ($C_9H_{13}O_4$), 167.0717 ($C_9H_{11}O_3$), 141.0924 ($C_8H_{13}O_2$), 123.0812 ($C_8H_{11}O$), 111.0450 ($C_8H_7O_2$), 99.0454 ($C_5H_7O_2$), 81.0348 (C_5H_5O), 71.0142 ($C_3H_3O_2$), 57.0348 (C_3H_5O)
$C_{20}H_{30}O_6$ -1	365.1970	16.85	0.18	199.0974 ($C_{10}H_{15}O_4$), 183.1027 ($C_{10}H_{15}O_3$), 181.0870 ($C_{10}H_{13}O_3$), 141.0924 ($C_8H_{13}O_2$), 139.1127 ($C_9H_{15}O$), 123.0812 ($C_8H_{11}O$), 85.0297 ($C_4H_5O_2$), 73.0296 ($C_3H_5O_2$)
$C_{20}H_{30}O_6$ -2		16.23	0.03	199.0974 ($C_{10}H_{15}O_4$), 183.1027 ($C_{10}H_{15}O_3$), 181.0870 ($C_{10}H_{13}O_3$), 139.1127 ($C_9H_{15}O$), 125.0973 ($C_8H_{13}O$), 97.0662 (C_6H_9O), 57.0348 (C_3H_5O)
$C_{20}H_{30}O_6$ -3		16.35	0.05	199.0974 ($C_{10}H_{15}O_4$), 183.1027 ($C_{10}H_{15}O_3$)
$C_{20}H_{30}O_7$ -1	381.1912	14.58	0.06	199.0974 ($C_{10}H_{15}O_4$), 185.0819 ($C_9H_{13}O_4$), 181.0870 ($C_{10}H_{13}O_3$), 153.0916 ($C_9H_{13}O_2$), 141.0924 ($C_8H_{13}O_2$), 137.0975 ($C_9H_{13}O$), 123.0812 ($C_8H_{11}O$),

				85.0297 ($C_4H_5O_2$), 73.0296 ($C_3H_5O_2$), 59.0138 ($C_2H_3O_2$)
$C_{20}H_{30}O_7$ -2		18.09	0.03	199.0974 ($C_{10}H_{15}O_4$), 185.0819 ($C_9H_{13}O_4$), 181.0870 ($C_{10}H_{13}O_3$), 157.0874($C_8H_{13}O_3$), 153.0916 ($C_9H_{13}O_2$), 139.0763 ($C_8H_{11}O_2$), 73.0296 ($C_3H_5O_2$), 59.0138 ($C_2H_3O_2$)
$C_{20}H_{30}O_7$-3		17.06	0.06	
$C_{20}H_{32}O_6$-1	367.2125	20.69	0.35	183.1027 ($C_{10}H_{15}O_3$), 157.0874($C_8H_{13}O_3$), 139.0766 ($C_8H_{13}O_2$), 59.0138 ($C_2H_3O_2$)
$C_{20}H_{32}O_6$-2		23.42	0.02	199.0974 ($C_{10}H_{15}O_4$), 157.0874($C_8H_{13}O_3$), 59.0138 ($C_2H_3O_2$)
$C_{20}H_{32}O_6$-3		16.05	0.03	199.0974 ($C_{10}H_{15}O_4$), 185.0819 ($C_9H_{13}O_4$), 167.0717 ($C_9H_{11}O_3$), 141.0924 ($C_8H_{13}O_2$), 137.0973 ($C_9H_{13}O$), 123.0812 ($C_8H_{11}O$), 111.0452 ($C_6H_7O_2$), 81.0347 (C_5H_5O), 71.0142 ($C_3H_3O_2$), 57.0348 (C_3H_5O)
$C_{20}H_{32}O_7$-1	383.2074	18.98	0.04	
$C_{20}H_{32}O_7$ -2		16.1	0.12	
$C_{20}H_{32}O_8$-1	399.2021	18.25	0.11	241.1080 ($C_{12}H_{17}O_5$), 199.0974 ($C_{10}H_{15}O_4$), 183.1027 ($C_{10}H_{15}O_3$), 181.0870 ($C_{10}H_{13}O_3$), 167.0717 ($C_9H_{11}O_3$), 141.0924 ($C_8H_{13}O_2$), 135.0814 ($C_9H_{11}O$), 123.0812 ($C_8H_{11}O$), 113.0610 ($C_6H_9O_2$), 85.0297 ($C_4H_5O_2$), 71.0138 ($C_3H_3O_2$), 59.0138 ($C_2H_3O_2$), 57.0348 (C_3H_5O)
$C_{20}H_{32}O_8$-2		16.64	0.05	241.1080 ($C_{12}H_{17}O_5$), 199.0974 ($C_{10}H_{15}O_4$), 185.0819 ($C_9H_{13}O_4$), 183.1026 ($C_{10}H_{15}O_3$), 153.0916 ($C_9H_{13}O_2$), 141.0925 ($C_8H_{13}O_2$), 125.0971 ($C_8H_{13}O$), 113.0610 ($C_6H_9O_2$), 95.0504 (C_6H_7O), 69.0347 (C_4H_5O), 59.0139 ($C_2H_3O_2$), 57.0348 (C_3H_5O)

Supplement References:

(1) Ye, Q.; Wang, M.; Hofbauer, V.; Stolzenburg, D.; Chen, D.; Schervish, M.; Vogel, A.; Mauldin, R. L.; Baalbaki, R.; Brilke, S.; et al. Molecular Composition and Volatility of Nucleated Particles from α-Pinene Oxidation between −50 °C and +25 °C. *Environmental Science & Technology* **2019**, *53* (21), 12357-12365. DOI: 10.1021/acs.est.9b03265.
(2) Orlando, J. J.; Tyndall, G. S. Laboratory studies of organic peroxy radical chemistry: an overview with emphasis on recent issues of atmospheric significance. *Chemical Society Reviews* **2012**, *41* (19), 6294-6317, 10.1039/C2CS35166H. DOI: 10.1039/C2CS35166H.
(3) Ziemann, P. J.; Atkinson, R. Kinetics, products, and mechanisms of secondary organic aerosol formation. *Chemical Society Reviews* **2012**, *41* (19), 6582-6605, 10.1039/C2CS35122F. DOI: 10.1039/C2CS35122F.
(4) Zhang, X.; McVay, R. C.; Huang, D. D.; Dalleska, N. F.; Aumont, B.; Flagan, R. C.; Seinfeld, J. H. Formation and evolution of molecular products in α-pinene secondary organic aerosol. *Proceedings of the National Academy of Sciences* **2015**, *112* (46), 14168-14173. DOI: doi:10.1073/pnas.1517742112.
(5) Tiszenkel, L.; Stangl, C.; Krasnomowitz, J.; Ouyang, Q.; Yu, H.; Apsokardu, M. J.; Johnston, M. V.; Lee, S. H. Temperature effects on sulfuric acid aerosol nucleation and growth: initial results from the TANGENT study. *Atmos. Chem. Phys.* **2019**, *19* (13), 8915-8929. DOI: 10.5194/acp-19-8915-2019.
(6) Tiszenkel, L.; Lee, S.-H. Synergetic Effects of Isoprene and HOx on Biogenic New Particle Formation. *Geophysical Research Letters* **2023**, *50* (14), e2023GL103545. DOI: https://doi.org/10.1029/2023GL103545.
(7) Tiszenkel, L.; Pedersen, A. N.; Vasudevan-Geetha, V.; Hopf, M. C.; Glasius, M.; Elm, J.; Lee, S.-H. Oxygenated Organosulfates Are an Effective Nucleation Precursor in Mixed Biogenic and Anthropogenic Environments. *Geophysical Research Letters* **2026**, *53* (1), e2025GL117259. DOI: https://doi.org/10.1029/2025GL117259.
(8) Demarque, D. P.; Crotti, A. E. M.; Vessecchi, R.; Lopes, J. L. C.; Lopes, N. P. Fragmentation reactions using electrospray ionization mass spectrometry: an important tool for the structural elucidation and characterization of synthetic and natural products. *Natural Product Reports* **2016**, *33* (3), 432-455, 10.1039/C5NP00073D. DOI: 10.1039/C5NP00073D.
